# Scale-free flow of life: on the biology, economics, and physics of the cell

**DOI:** 10.1186/1742-4682-6-6

**Published:** 2009-05-05

**Authors:** Alexei Kurakin

**Affiliations:** 1 Department of Pathology, Beth Israel Deaconess Medical Center and Harvard Medical School, Boston, MA 02215, USA

## Abstract

The present work is intended to demonstrate that most of the paradoxes, controversies, and contradictions accumulated in molecular and cell biology over many years of research can be readily resolved if the cell and living systems in general are re-interpreted within an alternative paradigm of biological organization that is based on the concepts and empirical laws of nonequilibrium thermodynamics. In addition to resolving paradoxes and controversies, the proposed re-conceptualization of the cell and biological organization reveals hitherto unappreciated connections among many seemingly disparate phenomena and observations, and provides new and powerful insights into the universal principles governing the emergence and organizational dynamics of living systems on each and every scale of biological organizational hierarchy, from proteins and cells to economies and ecologies.

## Background

The introduction of proteomics technologies has opened unprecedented opportunities to compile comprehensive "parts lists" for various macromolecular complexes, organelles, and whole cells. In a typical proteomics experiment, an organelle or a macromolecular complex of interest, such as mitochondria [1,2], lysosomes [3], synaptosomes [4], postsynaptic densities [5,6], phagosomes [7], or lipid rafts [8-10], is purified from cultured cells or a tissue, using one of the available fractionation/isolation techniques. The protein components present in a given isolate are further dissociated and spatially resolved, typically by gel electrophoresis or chromatography. Finally, the identities of individual proteins are determined with the aid of mass spectrometry. A review of the multiple "parts lists" obtained for various organelles and complexes clearly shows that they share one noticeable pattern-they invariably feature proteins that are not expected to be present in the studied complex/organelle/location. Given the nature of sample preparation, potential cross-contamination during isolation procedures is always an issue in proteomics experiments. It is natural, therefore, that the surprises of apparent "mislocalization" revealed in proteomics experiments are commonly disregarded and ignored. Yet a number of investigators have pointed out that, at least in some cases, apparently "mislocalized" proteins cannot be easily explained away as cross-contaminants [7,9]. In addition, as proteomics data accumulate, certain recurring patterns in protein "mislocalization" begin to emerge. For example, various metabolic enzymes, particularly proteins involved in energy metabolism, such as F_1 _F_0 _ATP synthase components and glycolytic enzymes, have been found in diverse and seemingly unrelated cellular locations, complexes, and organelles [3,4,7-9,11]. Taken together, proteomics studies appear to suggest that protein localization in the cell may be inherently uncertain or, at least, significantly more flexible and dynamic than is commonly believed.

Surprise is a sign of failed expectations. Expectations are always derived from some basic assumptions. Therefore, any surprising or paradoxical data challenges either the logical chain leading from assumptions to a failed expectation or the very assumptions on which failed expectations are based. When surprises are sporadic, it is more likely that a particular logical chain is faulty, rather than basic assumptions. However, when surprises and paradoxes in experimental data become systematic and overwhelming, and remain unresolved for decades despite intense research efforts, it is time to reconsider basic assumptions.

One of the basic assumptions that make proteomics data appear surprising is the conventional deterministic image of the cell. The cell is commonly perceived and traditionally presented in textbooks and research publications as a pre-defined molecular system organized and functioning in accord with the mechanisms and programs perfected by billions years of biological evolution, where every part has its role, structure, and localization, which are specified by the evolutionary design that researchers aim to crack by reverse engineering. When considered alone, surprising findings of proteomics studies are not, of course, convincing enough to challenge this image. What makes such a deterministic perception of the cell untenable today is the massive onslaught of paradoxical observations and surprising discoveries being generated with the help of advanced technologies in practically every specialized field of molecular and cell biology [12-17].

One of the aims of this article is to show that, when reconsidered within an alternative framework of new basic assumptions, virtually all recent surprising discoveries as well as old unresolved paradoxes fit together neatly, like pieces of a jigsaw puzzle, revealing a new image of the cell–and of biological organization in general–that is drastically different from the conventional one. Magically, what appears as paradoxical and surprising within the old image becomes natural and expected within the new one. Conceptually, the transition from the old image of biological organization to a new one resembles a gestalt switch in visual perception, meaning that the vast majority of existing data is not challenged or discarded but rather reinterpreted and rearranged into an alternative systemic perception of reality. To appreciate the new image of biological organization and its far-reaching ramifications, let us overview various experimental surprises and paradoxes, while watching how seemingly unrelated and incompatible pieces fall together into one self-consistent and harmonious picture.

## Ambiguity in protein localization, interactions, structure, and function

Large-scale studies of protein-protein interactions have unexpectedly revealed that the typical number of interactors for a given protein is far greater than our textbook-nurtured intuition would expect [17-23]. Importantly, the identified interactors of a given protein are often dispersed among diverse macromolecular complexes and cellular locations. In the same way and largely for the same reasons as in the case of surprising proteomics data, a researcher with conventional deterministic views on cellular organization normally disregards those potential interactors that are not expected to co-reside with a protein of interest in the same cellular location. In fact, the contrast between the habitual deterministic perception of the cell and the apparently promiscuous nature of protein interactions implied in large-scale protein interaction studies is so obvious and unsettling that it has triggered a flurry of publications questioning and analyzing the reliability of large-scale protein interaction studies and the results they generate [24-27]. Yet it is not difficult to see that the paradox of "promiscuous" protein interactions can be resolved simply by entertaining a more dynamic, flexible, and inherently probabilistic view on the partitioning of proteins inside the cell. Breaking away from the conventional deterministic perception of cellular organization opens an opportunity to interpret multiple interactions detected in large-scale studies as potentialities that may be and, perhaps, are realized, even if transiently, under certain circumstances, in certain locales, and/or in certain times. This is not to say, of course, that there are no spurious hits in large-scale protein interaction data, but to suggest that there may be far fewer of them than the habit of perceiving cellular organization as pre-determined allows one to accept as believable.

As usual, reality is in harmony with itself, for the biophysical basis of inherent ambiguity in protein-protein interactions is being revealed in a continuous series of surprising discoveries in the field of protein science. The detailed, colorful, but static images of proteins that populate textbooks and the covers of biological publications inadvertently reinforce the old and misleading perception of proteins as deterministic "building blocks and machines of the cell". The latest experimental evidence attests that nothing could be further from the truth. "Dynamics", "ambiguity", and "adaptive plasticity" are becoming the key words in the description of protein structure and function [17,28,29]. Progress in research technology and methods, together with the advances in our understanding of protein biophysics, are bringing about a novel image of the protein as a dynamic and adaptive molecular organization [28,30-33].

**Figure 1:**
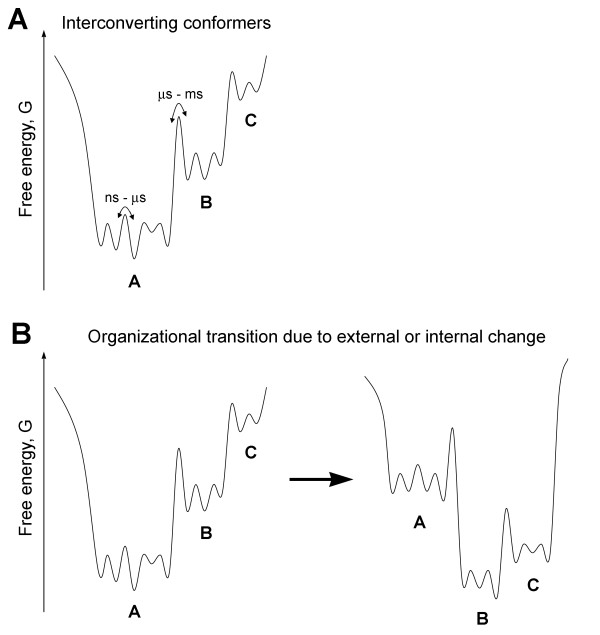
**The concept of protein conformational landscape**. **A**) Any protein structure exists in solution as a population of interconverting conformers, shown here as minima on the free energy curve, which represents a one-dimensional cross-section through the high-dimensional energy surface of a protein. In the example given, a population of conformers is composed of three families (A, B, and C). Families are composed of groups of related conformers, while groups, in turn, are composed of yet smaller divisions (not shown). The rates of interconversions are defined by the energy barriers separating alternative conformations. Interconversions on timescales of microseconds and slower usually correspond to large-scale collective (domain) motions within the protein structure, which are relatively rare. Loop motions and side-chain rotations typically occur on timescales of pico- to microseconds, while atom fluctuations occur on timescales of picoseconds and faster. **B**) Changes in external (environmental) conditions (pH, temperature, pressure, ionic strength, etc.) or in the internal state of the protein (e.g. ligand binding, mutation, posttranslational modification) often lead to redistribution of protein conformers and altered rates of their interconversions, i.e. to a reshaping of protein conformational landscape.

Combining nuclear magnetic resonance spectroscopy and molecular dynamics simulations Lindorff-Larsen et al. showed that even the hydrophobic cores of tightly folded proteins behave more like liquids rather than solids [34]. Single molecule studies necessitated the introduction of such concepts as static and dynamic disorders, the former to reflect the fact that any population of seemingly identical (isogenic) protein molecules is always composed of different individuals and the latter to indicate that the properties of the same individual molecule change in time [35-37]. Any protein structure exists in solution as a population of conformer families. The protein structure continuously and stochastically samples its different conformations, undergoing relatively slow structural transitions between different families of related conformers and relatively fast transitions within a given conformer family [29,32] (Fig. 1). Moreover, the conformational landscape of the protein is not fixed. Binding of ligands, posttranslational modifications, temperature, pressure, solvent and other factors may drastically alter the conformational landscape by triggering a redistribution of conformers and changing heights of the energy barriers separating alternative conformers [29,38,39] (Fig. 1B). Because different conformers can potentially bind different ligands and perform different cellular functions, ambiguity in protein interactions, localization, and function is an inevitable and natural consequence of the conformational heterogeneity and structural plasticity of proteins [17,32].

Yet apparently even a statistical description of the protein structure wandering randomly through its pliable conformational landscape does not exhaust all the surprises that proteins keep in store for us. The latest studies addressing the structure and dynamics of various enzymes suggest that the walk of a protein structure through its conformational landscape is actually not random, but proceeds along statistically preferred routes that, strikingly enough, happen to correspond to the conformational changes observed during actual enzymatic catalysis [40-44]. In other words, a substrate-free enzyme prefers to sample the sequence of coupled conformational transitions that corresponds to actual changes in its structure when the enzyme performs its function.

For further discussion, it is worth pointing out that the conformational sequence "pre-sampled" by an enzyme in anticipation of catalysis constitutes, in essence, a "behavioral routine" (a form of memory) of the enzyme, which, conceptually, is not different from behavioral routines (procedural memories) of humans.

Human behavioral routines represent useful or adaptive activity patterns that are culled from among the relatively unorganized and rather chaotic motor-neuronal and cognitive activity in the course of individual development and learning. With time, behavioral routines become "hard-wired", i.e. probabilistically preferred, and are activated later in life automatically, normally outside of awareness (and sometimes out of context) [45]. Taking into account the fact that a protein's conformational landscape depends on environmental context and on the protein's own state (e.g., posttranslational modifications), one can envisage that different environments and different protein states may elicit different "behavioral routines" in the same protein. In other words, it is very likely that any given enzyme/protein possesses, in fact, a whole repertoire of context- and state-dependent behavioral routines rather than a single routine, the repertoire that has been "hard-wired" into protein structural dynamics as a set of useful sequences of coupled conformational transitions selected and "remembered" in the course of the co-evolution of a given enzyme/protein and its host. Pertinently, the existence of protein "behavioral repertoires" would provide an elegant explanation of how and why the same protein performs multiple and often unrelated functions within the cell or organism. As concrete examples, consider the mitochondrial enzyme, dihydrolipoamide dehydrogenase (DLD), a versatile oxidoreductase with multiple roles in energy metabolism and redox balance. Environmental conditions that destabilize the DLD homodimers reveal a hidden proteolytic activity of the oxidoreductase, turning it into a protease involved in the regulation of mitochondrial iron metabolism [46]. Myoglobin functions as a dioxygen storage protein at high pH, but as an enzyme in NO-related chemistry at low pH [47,48]. Aconitase, an enzyme of the tricarboxylic acid (TCA) cycle, loses its enzymatic activity when iron levels in the cytosol become too low and functions as an iron-responsive-element-binding protein that regulates the mRNAs encoding ferritin and the transferrin receptor [49].

In fact, a list of proteins performing multiple functions in the cell or organism is long and rapidly expanding [50]. For example, the Clf1p splicing factor participates in DNA replication [51]; proteosomal subunits [52] and PutA proline dehydrogenase [53] serve as transcription regulators; ribosomal proteins function in DNA repair [54]; the enzyme of phenylalanine metabolism, DcoH, acts as a transcriptional regulator [55]; and the glycolytic pathway enzyme phosphoglucose isomerase functions as a neuroleukin [56], as an autocrine motility factor [57], and as a differentiation factor [58]. Notably, at least seven of 10 glycolytic enzymes and at least seven of 8 enzymes of the TCA cycle have been reported to have more than one function, with glyceraldehyde-3-phosphate dehydrogenase (GAPDH) and its 10 confirmed non-enzymatic functions representing one of the champions in versatility [59,60]. Proteins performing multiple functions have come to be recognized as a phenomenon in itself under the cliché "moonlighting proteins" [50]. The phenomenon of moonlighting proteins remains an unexpected and unexplained oddity within the conventional image of cellular organization. Notice, however, that, in the light of the inherent ambiguity and adaptive plasticity of protein localization, interactions, and structure, the surprising discovery of multifunctional proteins becomes less paradoxical and even expected in hindsight.

An account of recent remarkable discoveries in the field of protein science would be incomplete without mentioning the so-called natively unfolded proteins–one of the extreme cases of protein adaptability, ambiguity, and disorder. Natively unfolded proteins remain unstructured in solution, when isolated from cellular environment. They acquire a defined structure only when complexed with other molecules [61-63]. The discovery of intrinsically disordered proteins has come as a total surprise, since the concept of natively unfolded proteins cannot be readily assimilated either within the conventional "structure-defines-function" paradigm of protein science or within the deterministic image of the cell. The structures and functions of naturally unfolded proteins are inherently contextual, i.e. defined in large measure by their microenvironment and interacting partners. Because a major fraction of eukaryotic proteins is predicted to have large, intrinsically disordered regions in their structures, and because these regions are apparently important for protein functions and interactions [61,63], the partitioning and organization of proteins inside the cell cannot rely on the specificity provided by protein structure alone, but should be driven by some unknown principles that are different from, and complementary to the conventional principles of molecular recognition expressed in the "lock-and-key" metaphor. Structurally ambiguous or even simply flexible proteins have *a choice*, since they can interact with different partners, join different macromolecular organizations, perform different actions, and contribute in different ways to the functioning of diverse macromolecular complexes and sub-cellular structures.

It should be also pointed out that the adaptive plasticity and ambiguity in protein structure and behavior are almost certain to be strictly enforced by natural selection, for they underlie adaptive plasticity at higher levels of biological organizational hierarchy [17,28]. Indeed, if proteins were deterministic or nearly deterministic entities, then the adaptability of their host cells and organisms would be severely compromised, being limited to the relatively long timescales on which the adaptation through genetic variation, selection, and heredity operates. The balance between order and disorder in protein structure, function, and interactions ensures that higher-order macromolecular complexes and sub-cellular structures, and thus vital cellular functions, remain flexible and adaptive on relatively short timescales that are too fast to involve genetic mechanisms and that require rapid and efficient epigenetic adaptations. It is fair to assume that those cells and organisms that fail to adapt on short timescales are quickly weeded out by natural selection in complex and dynamic environments where competition and change take place simultaneously on multiple timescales, ranging from extremely fast to extremely slow.

## Dynamic partitioning of proteins in living cells

The recent introduction of genetically encoded fluorescent tags, together with accompanying advances in imaging technologies and image processing, has allowed researchers to observe and analyze individual proteins and other molecules in real time within their natural environments, i.e. in living cells and tissues. Perhaps the most surprising discovery that has emerged from such studies is the unexpectedly high degree of dynamism observed within a wide variety of sub-cellular structures and macromolecular complexes. Studies addressing behavior of individual molecules in living cells show that many, and perhaps all, of the sub-cellular structures and macromolecular complexes once regarded as relatively stable are in fact highly dynamic, steady state molecular organizations (see [14,64,65] for reviews).

A classical example of steady state molecular organization is a treadmilling actin filament, which represents a continuous process of polymerization and depolymerization of actin monomers entering and leaving actin polymer at its ends with varying rates [14,66]. When the processes of polymerization and depolymerization are balanced in counteracting each other, actin filament maintains its length and its physical identity/appearance. If the counteracting processes of adding and shedding actin monomers are unbalanced, the actin filament grows or shrinks, appears or disappears. Quantitative visualization of individual fluorescently tagged components of various subcellular structures and complexes, combined with photobleaching experiments and computer-aided analysis and modeling, show that many macromolecular structures in the living cell are maintained as dynamic steady-state organizations, conceptually similar to treadmilling actin filament, but of a greater complexity. Examples include, but are not limited to, various nuclear compartments, such as nucleoli, Cajal bodies, promyelocytic leukemia (PML) bodies, splicing factor compartments, nuclear pore complexes and others, euchromatin, heterochromatin, the cytoskeleton, the Golgi complex, as well as the macromolecular holocomplexes mediating basic biological processes, such as DNA replication and repair machineries, transcription apparatus and others [14]. Remarkably enough, even elongation factors have been found in dynamic and rapid exchange between two molecular pools, the elongation factors transiently associated with the elongating RNA polymerase complexes and the freely diffusing pool of factor molecules in the nucleoplasm [67]. Steady-state macromolecular organizations are sustained by the flow of energy and matter passing through them, with their resident components entering and leaving organizations with widely different recruitment probabilities, residence times, and turnover rates [14,64,65,68].

**Figure 2:**
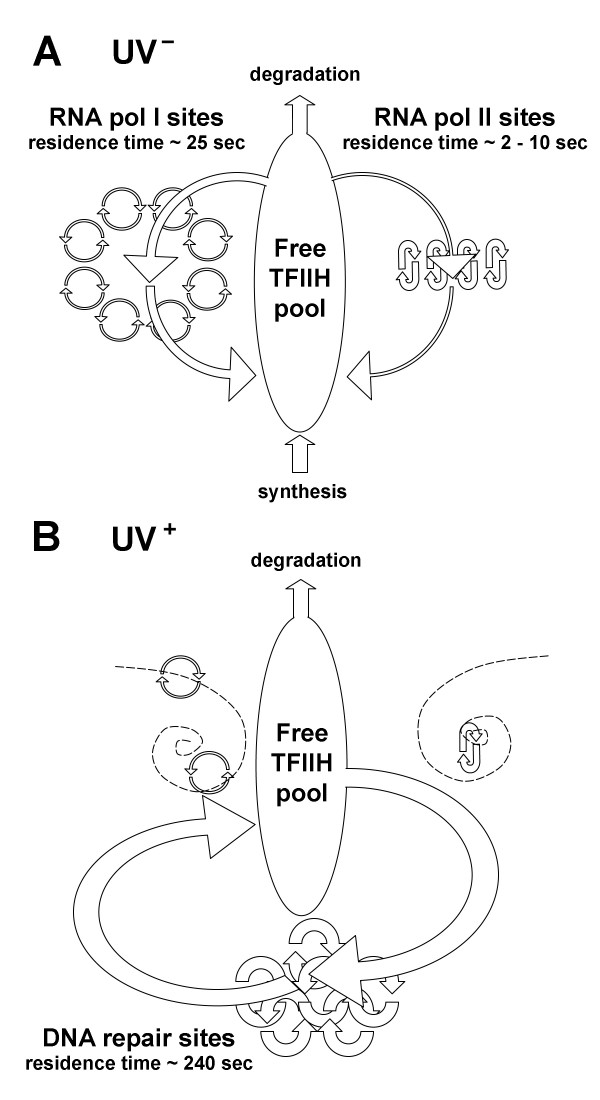
**Dynamic partitioning of TFIIH in the nucleoplasm**. Quantitative visualization and analysis of the fluorescently-tagged transcription factor IIH (TFIIH) molecules in living cells [69] suggest that TFIIH partitions dynamically among at least four distinct molecular pools in the nucleoplasm: a freely diffusing "unemployed" pool, RNA polymerase I and II transcription sites, and DNA repair sites. **A**) In the absence of DNA damage (UV^-^), the average residence times of TFIIH employed in transcription are approximately 25 and 5 seconds for the sites of RNA pol I and II, correspondingly. **B**) Upon DNA damage (UV^+^), TFIIH reversibly repartitions into DNA repair sites, where its average residence time is significantly longer, 240 seconds, while transcription ceases in the meantime. As the steady-state level of TFIIH in the cell is tightly controlled, the competitive partitioning of TFIIH between different functional pools may potentially couple and coordinate such cellular functions as transcription and DNA repair, both locally and globally. The dynamic partitioning of TFIIH is one of the concrete examples of how the fluxes of moonlighting activities, driven by essentially economic supply-and-demand-type relationships, can lead to a seamless and "design-free" integration of diverse cellular functions into one dynamic and adaptive functional whole that performs and evolves as a self-organizing molecular-scale economy.

In addition to the highly dynamic, steady state nature of sub-cellular structures and compartments, a number of other characteristic patterns have emerged from studies of molecular movement in living cells. First, proteins often dynamically partition between two or more macromolecular organizations, where they perform different and sometimes apparently unrelated cellular functions. As an example, the study by Hoogstraten et al. [69] shows that molecules of the transcription factor IIH (TFIIH) are continuously exchanged among at least four distinct pools inside the nucleus: the sites of RNA polymerase I transcription, the sites of RNA polymerase II transcription, DNA repair sites, and the freely mobile pool of TFIIH in the nucleoplasm (Fig. 2). The average residence time of TFIIH within a given pool is defined by the transient specific associations and activity of the TFIIH molecules within functional macromolecular complexes comprising the pool. In the absence of DNA damage, functional TFIIH localizes to the sites of transcription. However, induction of DNA damage leads to a dynamic and reversible redistribution of TFIIH, which accumulates at sites of DNA repair, where its average residence time is much longer. The extent and duration of TFIIH redistribution is proportional to the DNA damage load and lasts until damage has been repaired. To the extent that the processes of transcription and DNA repair compete with each other for the shared pool of TFIIH, they become interconnected and interdependent. It is worth pointing out that links between the various processes competing for TFIIH can potentially be made either stronger or weaker, simply by regulating the availability of TFIIH and its turnover in the nucleoplasm. Indeed, investigators found that the steady-state level of TFIIH is strictly controlled in the cell [69]. It is worth noting that, in network terms, the ability to regulate the strength of links allows a given network structure to combine and balance two critically important but mutually contradictory organizational properties: stability and plasticity.

The second notable pattern emerging from the studies on molecular behavior in living cells is that any given protein usually partitions into macromolecular organizations only when it is functionally competent. Inactive proteins tend to remain in a freely diffusing, "unemployed" pool and/or to have significantly shorter residence times within the molecular organizations employing them, as compared to their functionally competent copies [68,70].

Third, a protein may be recruited to a given macromolecular organization only temporarily, when its particular activity/competence is needed, and it is discharged into the freely mobile pool when its services are no longer required within the evolving macromolecular organization [67,69,71,72]. Symmetrically, but on a higher-order organizational scale, it appears that many, perhaps all, macromolecular complexes and sub-cellular structures are assembled and maintained as steady-state molecular organizations only when they perform their functions. They are dissolved or restructured when their functions are no longer needed or altered within the cell. This phenomenon manifests itself as a tight coupling between the architecture and function of sub-cellular compartments/complexes. Inhibition of ribosomal gene transcription results in disassembly of the nucleolus [73]. Conversely, the addition of extrachromosomal ribosomal genes leads to the appearance of micronucleoli [74,75]. Re-expression of the Cajal body resident p80-coilin protein in p80-knockout cells is sufficient to regenerate Cajal bodies [76]. Blocking the efflux of splicing factors from splicing compartments leads to the enlargement and reshaping of the latter [64]. Nuclear and other intracellular compartments are naturally lost and re-assembled during the course of each cell division [77,78].

Taken together, the results of the studies addressing molecular dynamics in living cells indicate that sub-cellular structures and macromolecular complexes are formed in response to the functional needs of the cell, in a self-organized manner. They are dynamically maintained as steady-state organizations while performing their functions, and they are dissolved when their functions are no longer required [14,64]. Since the functional needs of the cell surviving in unpredictable and competitive environments continuously change on multiple scales of space and time, it is reasonable to suggest that self-organization of diverse intracellular compartments, structures, and complexes is driven by changing priorities and demands of the evolving and adapting cellular economy. The continual turnover and re-organization, achieved through competitive partitioning of proteins and other molecules into transient steady-state macromolecular organizations that form and dissolve in response to the continuously changing needs of the cellular economy, represent then a unending process meant to optimize the balance between two opposites: on the one hand, economic efficiency, which requires adequate and stable organization; and on the other hand, adaptability, which requires organizational flexibility and change. In fact, striking a proper balance between efficiency and adaptability is a necessary pre-requisite for the competitive performance of organizations and economies at each and every scale of biological organizational hierarchy, from molecules, cells, and organisms to business enterprises and national economies [79].

It is also worth pointing out that the economic conceptualization of cellular organization implies that the integration of diverse sub-cellular structures and macromolecular complexes into one coordinated whole of the cell is achieved in a self-organized and self-regulated manner, i.e. without any external architect or design. The competitive partitioning and exchange of shared molecular components among functionally and structurally distinct sub-cellular compartments, structures, and complexes represents an optimizational strategy that ensures integration, coordination, and efficiency, but, at the same time, allows for rapid and flexible organizational adaptations. It is worth noting that such an interpretation of cellular organization transforms many seemingly unrelated and paradoxical discoveries generated in various specialized fields of molecular and cell biology into harmoniously interconnected and interrelated parts of one and the same image, namely that of the cell living and evolving as a self-organizing and self-regulating molecular-scale economy.

One of the first questions that the economic interpretation of the cell may raise is where and how such a well-known "economic" aspect of cellular activity as metabolism fits into the picture.

## Dynamic compartmentalization and substrate channeling in cellular metabolism

Broadly defined, "compartmentalization of metabolism" traditionally refers to an ordered physical association or clustering of metabolic enzymes performing sequential steps in a given metabolic pathway. "Substrate channeling" denotes a relative isolation of metabolic intermediates from the bulk cytoplasm within a macromolecular organization of compartmentalized enzymes [80,81]. In an ideal arrangement, all enzymes of a given metabolic pathway are assembled into a stable multienzyme complex in which metabolic intermediates, isolated from the bulk cytoplasm, are passed along a physical channel/tunnel connecting active sites arranged in a sequence. Such an organization allows for rapid and efficient production with little dissipation [82-85]. It is useful to note that, given efficient internal transport and conversions, the rate of metabolic flux through an ideally organized multienzyme complex is not limited by diffusion but by the rate of delivery of the first substrate to the complex and by the rate of consumption of the last product leaving the complex. The more organized and coordinated are the individual enzymes in a complex or compartment, the less relevant diffusion becomes for the rate of metabolic production. Increasingly looser organization/coordination makes diffusion increasingly more relevant and unproductive energy/matter dissipation more significant.

From both evolutionary and economic perspectives, the organization and compartmentalization of metabolism seem natural and inevitable, for cells competing for limited amounts of shared resources are forced to survive under the constant and often severe evolutionary pressure to minimize dissipation of energy and matter within their internal economies, while maximizing metabolic production and its efficiency. As our human-scale experience with economic systems suggests, maximization of production and its efficiency can be achieved only through division of labor and spatiotemporal organization of production and exchange. In addition, since metabolic intermediates are often limiting, unstable, and sometimes toxic, compartmentalization and substrate channeling may become essential if only to ensure the survival of producers.

Unfortunately, the early *in vitro *studies demonstrating the existence of stable metabolic compartments and substrate channeling did not seem convincing or generalizable enough to overcome the long-held tradition in mainstream biochemistry that treats the cell as a biochemical reactor of well-mixed and freely diffusing reactants. As traditional views slowly yield to the onslaught of experimental evidence exemplified by the discoveries of purinosomes [86], transamidosomes [87], carboxysomes [88], glycosomes [89,90], the branched amino acid metabolon [91], dhurrin biosynthesis metabolon [92], and other "-somes" and metabolons, it is useful to summarize the recurring themes and patterns emerging from the large body of experimental literature on metabolic organization [80,81,93-100].

First of all, the phenomenon of metabolic compartmentalization appears to be evolutionarily conserved. It has been observed in bacteria [88], yeast [101], plants [98,102], and mammals [86]. However, in contrast to conventional cellular compartments, which are relatively stable and are present in most cells most of the time under most conditions, metabolic compartments are often assembled on demand to satisfy changing or local needs of cellular economy that emerge in response to transitory environmental challenges and opportunities.

Using fluorescently tagged individual enzymes, An et al. have recently shown that all six enzymes of the *de novo *purine biosynthetic pathway reversibly co-cluster in human cultured cells under purine-depleted conditions, but remain disorganized within the cytoplasm in purine-rich medium [86]. The formation of bacterial carboxysomes, polyhedral organelles consisting of metabolic enzymes encased in a multiprotein shell, is induced by low levels of CO_2_. The carboxysome improves the efficiency of carbon fixation by concentrating carbon dioxide and delivering it to ribulose biphosphate carboxylase/oxygenase, which resides in the lumen of the organelle and catalyzes the CO_2 _fixation step of the Calvin cycle [88]. The so-called *pdu *organelles, which are similar in shape and size to carboxysomes, are formed during growth of bacteria on 1, 2- propanediol (1, 2-PD) but not during growth on other carbon sources. Genetic studies suggest that the *pdu *organelles minimize the harmful effects of propionaldehyde, a toxic intermediate of 1, 2-PD degradation [103,104]. In plant cells, glycolytic enzymes have been reported to reversibly partition from a soluble pool to a mitochondria-bound pool upon increased respiration and back into the soluble pool upon inhibition of respiration. Mitochondrially-associated enzymes form a functional glycolytic sequence that supports mitochondrial respiration through substrate channeling, as revealed by NMR spectroscopy tracing of ^13^C-labeled precursors [98]. Notably, the increased demand for pyruvate consumption by respiring mitochondria is met through reversible partitioning and compartmentalization of glycolytic enzymes, rather than through the changes in their abundance. When rat cardiomyocytes are cultured in creatine-deficient medium, regularly shaped inclusions highly enriched in creatine kinase (CK) form inside their mitochondria. The emergence of these inclusions correlates with low levels of total intracellular creatine and can be reversed simply by adding creatine to the culture medium. The CK-rich mitochondrial inclusions are thought to be macromolecular complexes that form as a result of metabolic adaptation intended to speed up phosphocreatine production in order to keep up with intracellular demand for phosphocreatine when creatine levels are low [105].

It is clear from these and many other examples that metabolic compartments are often formed in a transient and reversible manner, in response to specific environmental challenges and opportunities. It can even be generalized that any environmental change normally triggers the formation and stabilization of metabolic compartments or complexes that self-organize either to alleviate the problems or to take advantage of the opportunities created by environmental change within the economy of the cell. There are obvious competitive advantages in a metabolic system that relies on dynamic redistribution and reorganization of metabolic enzymes, for such a system allows for a practically infinite variety of rapid and efficient metabolic responses, solutions, and adaptations to a potentially infinite diversity of environmental challenges, opportunities, and changes.

Such a dynamic image of metabolic organization is well supported experimentally in the particular case of glycolysis, a classical metabolic pathway used for intracellular production of energy in the form of ATP. Studies on spatiotemporal organization of glycolysis show that the glycolytic sequence functions as transiently immobilized enzymatic clusters associated with F-actin, cell membranes, and other molecular scaffolds [81,96,97,105-107]. The combinatorial versatility and spatiotemporal complexity of the glycolytic sequence come from i) the segmented nature of the glycolytic sequence, with individual segments able to function independently in response to specific metabolic demands; ii) the existence of multiple glycolytic enzyme isoforms differing in their binding properties to each other and/or to their scaffolds and regulatory molecules; and iii) the existence of multiple types and isoforms of scaffolding and regulatory molecules. The adaptive plasticity of the glycolytic sequence, which has evolved to meet an enormous diversity of specific energy demands varying on multiple scales of space and time within the organism and cell, relies on recurring organizational transitions. Such transitions involve transient relaxation of pre-existing arrangements of the sequence into a state of relative disorder, followed by the re-assembly of the sequence into new configurations and/or in new cellular locations in accord with changing metabolic demands [96].

What is true for glycolysis is likely to be true for all other metabolic pathways and for the metabolic system of the cell as a whole. In this regard, it is useful to briefly mention the main conclusions of recent graph-theoretical studies on metabolic organization [108-110]. Metabolic organization of the cell can be mathematically captured and analyzed in terms of a graph or network of interconnected chemical transformations, where nodes are metabolites and links are enzymes catalyzing the corresponding transformations. A graph-theoretical analysis of global metabolic networks in 43 different organisms shows that all metabolic systems are organized and maintained in the course of biological evolution as "small-world" scale-free networks [108,110]. This means that i) any chemical transformation or metabolite in the cell is a very small number of steps away from any other transformation or metabolite, respectively; and ii) even though many metabolites are involved in relatively few chemical transformations, a significant number of metabolites participate in a great variety of metabolic pathways and reactions, as reflected in the fact that the number of links per node in metabolic networks follows a power law [108]. It is extremely difficult, and perhaps impossible, to imagine how scale-free connectivity in metabolic organization could have evolved or be maintained inside the cell without metabolic compartmentalization and substrate channeling. It is also extremely difficult, and perhaps impossible, to imagine how scale-free metabolic organization can exist and function as a pre-defined and fixed system of metabolic compartments and substrate channels in conditions of constantly changing and unpredictable environments. In contrast, dynamic and reversible partitioning of enzymes into transient steady state metabolic compartments, which are continuously formed and disbanded in response to unpredictably changing metabolic demands, appears to be a natural solution that has appropriate analogies at the scale of human organizations and economies. From this perspective, it becomes less surprising that cellular protein interaction and metabolic networks share power-law scaling with a number of economic phenomena. Power-law scaling is a symptom of self-organized complexity. It is shared by many biological, economic, social, and certain physical phenomena, but it is not normally found in engineered constructions built according to a pre-conceived design [109,111].

As a whole, the research on metabolic organization suggests that cellular metabolic enzymes and metabolites continuously and dynamically partition between a solution phase circulating throughout the cell interior and a dynamic soft-matter phase existing in the form of a heterogeneous complex matrix made up of interdependent and interconnected molecular organizations/compartments that continuously change in size, composition, and relationships with one another on multiple scales of time and space. Individual metabolic compartments are integrated into one whole of the cellular economy through continuous and competitive partitioning of shared molecular components among diverse metabolic compartments. It should be noted that whether metabolic compartments are of a steady-state nature has not been studied systematically, because appropriate technologies and interest in mainstream research have been lacking. The recent studies, in which appropriate observations and measurements have been performed, suggest that metabolic compartments behave as highly dynamic, steady-state molecular organizations [86,112], in other words, like all other sub-cellular structures and macromolecular complexes scrutinized recently with the help of fluorescent microscopy and photobleaching techniques. It should be pointed out that, because many metabolic compartments are meant to satisfy cellular economic/metabolic demands that change rapidly in space and time, the majority of metabolic compartments are likely to be much more dynamic and much smaller than the relatively stable sub-cellular structures and macromolecular complexes meant to meet constant or slowly changing cellular needs, such as chromatin maintenance or macromolecular synthesis, processing, sorting, and trafficking. As a consequence, it is likely that due to their transient nature and small size, most metabolic compartments remain beyond the resolving power of techniques commonly used to analyze molecular dynamics in living cells. Needless to say, isolating a transient metabolic compartment for biochemical analysis is, in most cases, like picking up an eddy from a spring to have a closer look at its structure: one is always left with only water slipping between the fingers.

Summarizing, it can be concluded that the overall picture of cellular metabolic organization is conceptually identical to the dynamic image of sub-cellular organization revealed in living cells by modern fluorescence-based imaging technologies [14,64]. In fact, it is not difficult to see that these two images represent interrelated parts of one and the same image, with individual parts simply referring to different spatiotemporal scales. Specifically, one can suggest that all the well-known relatively large and stable sub-cellular structures and macromolecular complexes constitute the relatively higher levels in the hierarchy of cellular metabolic organization. In other words, they represent the macromolecular organizations that operate and change on relatively large and slow spatiotemporal scales, akin to large-scale social and business organizations and institutions in a national economy. On the other hand, what has been traditionally regarded as metabolic compartments and sequences represent molecular organizations matching and responding to changes taking place on relatively small and fast scales of space and time, akin to start-up companies, small firms, departments of large organizations and novel emerging businesses and institutions in a national economy. Metabolic compartments and sequences form and dissociate continuously, engaging in transient associations with various larger-scale sub-cellular structures and macromolecular complexes. Such transient associations ensure that the larger-scale sub-cellular structures and complexes functioning and evolving on relatively large and slow spatiotemporal scales are appropriately supplied with the specific forms of energy/matter that they require at different moments in time or in different locations in space. In other words, all the larger-scale sub-cellular structures and macromolecular complexes are built on, and, at the same time, support productive activity of various dynamic metabolic compartments/sequences that transiently associate with them through mutually profitable exchanges of energy/matter. Notice, that, such a perspective on cellular organization eliminates a conceptual divide between metabolism per se and any cellular structure or functional system. In other words, *the cell is a multi-scale continuum of metabolism–an economy*. Whatever molecule, complex, structure, or process we choose to consider, they all have some metabolic function within the hierarchically structured continuum of cellular economy, where they both define and are defined by metabolism. In precisely the same way, various human social and business organizations both define and are defined by the evolving economic system they form. Notice that such an image of the cell immediately resolves a panoply of paradoxes, such as the surprising ubiquity of glycolytic enzymes and the astonishing number of the different and seemingly unrelated functions they perform, or, as another example, why virtually all posttranslational modifications, currently more than 200, that mediate cellular epigenetic responses/adaptations involve products of basic metabolism (e.g. phosphorylation (ATP), methylation (S-adenosyl-methionine), acetylation (acetyl-CoA), ADP-ribosylation (NAD^+^), glycosylation (glucose), *O*-GlcNAcylation (UDP-GlcNAc), farnesylation (farnesyl pyrophosphate), palmitoylation (palmitic acid), arginylation (arginine), tyrosination (tyrosine), glutamylation (glutamate), and glycylation (glycine)).

At this point in our discussion, an attentive reader may point out that economics is a rather soft science, and of questionable predictive power, whereas molecular and cellular biology is assumed to be firmly rooted in physics, one of the most precise and reliable of sciences. The next natural question to be addressed, therefore, is how does the economic perspective on cellular organization relate to the mother of all modern sciences?

## The physics and metaphysics of dynamic compartmentalization

Indeed, since all cellular components, including small molecules, proteins, macromolecular complexes, sub-cellular structures, and the cell as a whole, are, first and foremost, physicochemical systems, it is imperative to make sure that physics, biology, and economics are in harmony and do not clash with one another within the image of the cell functioning as a self-organizing multiscale molecular economy.

Unfortunately, the basic courses of physics traditionally taught to biologists, such as classical mechanics and equilibrium thermodynamics–which have come to define for biologists what the pertinent physics is–are of little or no relevance for biology, for linearity and equilibrium have no place in living organisms and organizations, except maybe after their death. Any biological organization represents a far-from-equilibrium physicochemical process sustained by a continuous flow of energy/matter passing through the biological organization. Such processes are a subject of nonequilibrium thermodynamics and nonlinear physics, which are not included in the conventional biological curriculum.

Even though nonequilibrium thermodynamics is a relatively underdeveloped field, physicists studying simple nonequilibrium systems have generated over the years a wealth of useful concepts, observations, and empirical generalizations that can be quite illuminating when applied to biological and economic phenomena and systems. Therefore let us briefly review their basic findings.

**Figure 3:**
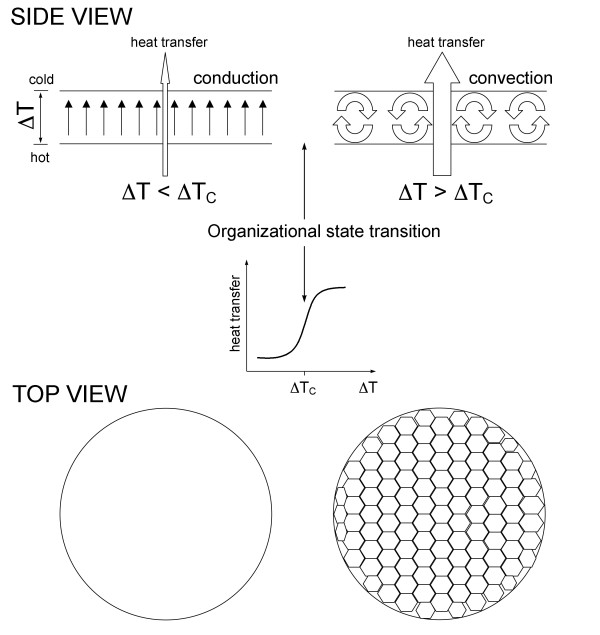
**The Benard instability**. Establishing an increasing vertical temperature gradient (ΔT) across a thin layer of liquid leads to a heat transfer through the layer by conduction (organizational state/form #1). Upon reaching a certain critical value of temperature gradient (ΔT_C_), an organizational transition takes place within the liquid layer and conduction is replaced by convection (organizational state/form #2), leading to a stepwise increase in the rate of heat transfer through the layer. The organizational state/form #2 (convection) is a more ordered state (higher negative entropy) than the organizational state/form #1 (conduction). The organizational state/form #2 (convection) will relax into the organizational state/form #1 (conduction) upon decreasing temperature gradient (not shown). As discussed in the text, the Benard instability is an example of a nonequilibrium dynamic system illustrating a number of the universal features shared by all biological (broadly defined) organizations: i) the emergence, maintenance, and development of any biological organization requires a continuous and accelerating flux of energy/matter through biological organization; ii) increasing the rate of energy/matter flux through a biological organization allows for growth in size and/or complexity; iii) any biological organization develops from states of relatively low order (low negative entropy) to states of relatively high order (high negative entropy); iv) increasing the rate of energy/matter flow through a biological organization leads to stepwise organizational state transitions and the emergence of organizational hierarchies and order that cover increasingly larger spatiotemporal scales; v) decreasing the rate of energy/matter flow through a biological organization leads to a stepwise hierarchical relaxation of ordered organization to states of lower negative entropy, and, eventually, to its dissolution and death (see more in the text).

Generating a gradient (e.g. temperature, concentration, chemical) within a relatively simple physicochemical system of interacting components normally causes a flux of energy/matter in the system and, as a consequence, the emergence of a countervailing gradient, which, in turn, may lead to the emergence of another flux and another gradient, and so on. The resulting complex system of conjugated fluxes and coupled gradients manifests itself as a spatiotemporal macroscopic order spontaneously emerging in an initially homogeneous system of microscopic components, provided the system is driven far enough away from equilibrium [113,114]. One of the classical examples of nonequilibrium systems is the Belousov-Zhabotinsky (BZ) reaction, in which malonic acid is oxidized by potassium bromate in dilute sulfuric acid in the presence of a catalyst, such as cerium or manganese. By varying experimental conditions, one can generate diverse ordered spatiotemporal patterns of reactants in solution, such as chemical oscillations, stable spatial structures, and concentration waves [114,115]. Another example is the Benard instability (Fig. 3). In this system, a vertical temperature gradient, which is created within a thin horizontal layer of liquid by heating its lower surface, drives an upward heat flux through the liquid layer. When the temperature gradient is relatively weak, heat propagates from the bottom to the top by conduction. Molecules move in a seemingly uncorrelated fashion and no macro-order is discernable. However, once the imposed temperature gradient reaches a certain threshold value, an abrupt organizational transition takes place within the liquid layer, leading to the emergence of a metastable macro-organization of molecular motion. Molecules start moving coherently, forming hexagonal convection cells of a characteristic size. As a result of the organizational transition, conduction is replaced by convection and the rate of energy/matter transfer through the layer increases in a stepwise manner.

Several empirical generalizations/laws obtained in studies of far-from-equilibrium systems are especially relevant for biology. First, a sufficiently intense flow of energy/matter through an open physicochemical system of interacting components *naturally *leads to the emergence of interdependent fluxes and gradients within the system, with concomitant dynamic compartmentalization of the system's components in space and time. Second, the emergence of macroscopic order is, as a rule, a highly nonlinear, cooperative process. When a critical threshold value of flow rate is exceeded, the system spontaneously organizes itself by partitioning its components into interdependent and interconnected steady state macroscopic organizations.

Importantly, what is preserved on the scales characteristic for such steady state macro-organizations are the spatiotemporal relationships between individual components, i.e. a certain organizational structure–*a form*–but not individual components passing through a given organization. Members come and go, but the organization persists. Third, varying experimental conditions, such as rates of influx and/or efflux of individual components, may lead to the emergence of distinct organizational configurations within the same set of interacting components/reactants. In other words, in far-from-equilibrium conditions, the same set of interacting components may form several, and potentially numerous, metastable organizational configurations, which are separated from each other by energetic barriers of different heights. The heights of energetic barriers define the probabilities of transitions between different organizational configurations; the barriers themselves are defined by the interplay between the internal dynamics of the system and external (environmental) influences. It is not difficult to see that the concepts of conformers (i.e. alternative metastable organizational states) and conformational landscape, introduced to describe the dynamics of protein structure (Fig. 1) are in fact scale-invariant, i.e. universal. They can be applied to describe the organization and dynamics of proteins, cells, organisms, business organizations, economies, ecosystems, and other open nonequilibrium systems comprising interacting components that continuously obtain, transform, and exchange different forms of energy/matter.

Perhaps the most important message for biology from the physics of nonequilibrium systems is that the emergence of gradients and spatial compartmentalization of molecules is a *common and natural *occurrence in a system of interacting molecules maintained in far-from-equilibrium conditions. As an open nonequilibrium physicochemical system, the cell is thus expected to exist as a complex, metastable organization of conjugated fluxes, steady-state compartments, and interdependent gradients. Notice, however, that conventional education and training leave no choice for biochemists and biologists but to treat intracellular compartments and gradients in terms of equilibrium thermodynamics and classical mechanics. It is not surprising therefore that the cell has come to be perceived as a well-mixed bag of reagents, where concentration- and diffusion-driven chemical transformations take place. It is not surprising therefore that, in order to account for experimentally observed intracellular gradients, compartments, and microenvironments, and in order to communicate their findings to one another and to the public, classically trained molecular and cell biologists have had to come up with such mechanistic notions as impermeable and semi-permeable membranes, pumps, channels, transporters, and motors. What one sees is defined by that what one knows [116]. In their interpretations of biological phenomena, most researchers have never moved beyond the conceptual frameworks of equilibrium thermodynamics and classical mechanics.

It is important to point out that the living cell has, in fact, a much greater capacity at self-organization than inorganic physicochemical systems commonly studied in nonequilibrium thermodynamics, because many cellular components, such as proteins, "know" and "remember" how to organize themselves. It is useful and conceptually correct to think about protein structure, and indeed any biological structure or organization, as a form of evolutionary memory [17]. Consider a metabolic enzyme, for example. As recent biophysical studies demonstrate, both the structure and inherent dynamics of an enzyme molecule "anticipate" recognizing and binding certain metabolites as well as performing on these metabolites certain actions that facilitate production of the chemicals/molecules that are likely to be in demand within the economy of the cell [40-44]. If this enzyme is normally a part of a multienzyme complex, its structure also "anticipates" functioning as a part of the multienzyme complex [117]. Because the same events, such as recognition, binding, catalysis, and functioning within a multiprotein organization, have been repeated again and again during the course of evolution, the memories of routine recognition, binding, catalysis, and collaboration have become embodied in the structure and dynamics of the enzyme. By generalizing this to all other proteins, it is not difficult to see that the self-organization of compartments, gradients, and fluxes within the cell is greatly facilitated and to a significant degree governed by evolutionary memory embodied in individual structures and dynamics of proteins. Notice that, superficially, the effect of evolutionary memory on cellular self-organization and dynamics, especially under stable and reproducible conditions, such as the ones routinely used in research laboratories, is reminiscent of design and determinism, and, naturally, will be interpreted as such by a mechanistically-minded person. There is a great deal of determinism in having breakfast every day, after all.

However, unlike the behaviors of parts in a machine, and similar to the behaviors of people in an economy, the structures and dynamics of proteins are not pre-determined by design but only statistically biased towards familiar recognition, interactions, and actions. Therefore, although being prone to functioning and forming multiprotein organizations "as usual" (following the economic principle of least effort), all proteins, and, consequently, the macromolecular organizations they form, remain flexible and open to adaptation, "learning", and evolution. As a consequence, having found itself in the situations or environments encountered frequently during the course of evolution, the cell "recognizes" a "familiar" situation by virtue of rapid self-organization of its proteins into those macromolecular complexes, compartments, and structures that proved to be useful for survival or prosperity in similar situations in the past. However, because cellular responses are inherently probabilistic, i.e. the cell always makes a choice among its alternative organizational configurations, which continuously compete with one another, the cell as an economy/organization remains flexible and adaptive, finding new responses/solutions to old situations/problems and "recognizing" new challenges and opportunities in its environment. In other words, the structure and dynamics of the cell, in precisely the same way as the structure and dynamics of the individual protein, are not pre-determined but only statistically biased towards familiar (learned) recognition, interactions, and actions. And in the same sense as the protein is an evolutionary memory, the cell represents an evolutionary memory too, but of a higher hierarchical order. It is not difficult to see that the same logic applies to and covers all scales of biological organizational hierarchy, from proteins and cells to tissues, organisms, organizations, economies, and ecosystems, leading us to the unavoidable conclusion that living matter as a whole is nothing else but a multi-scale continuum of evolving intelligence [79]. Such a conclusion is neither unexpected, nor is it counterintuitive: intelligence begets intelligence, machines beget only machines.

Returning to the physics of dynamic compartmentalization, nonequilibrium thermodynamics suggests a physical image of the cell that is drastically different from the accepted one. The emergence of intertwined fluxes, gradients, and steady-state compartments in nonequilibrium systems such as the cell occurs not because some molecules were designed to pump other molecules across semi-permeable barriers with the purpose of creating and maintaining concentration gradients – that is the inevitable and faulty logic of equilibrium thermodynamics and classical mechanics – but rather because a steady-state system of interdependent fluxes and gradients is a *normal *state of an open physicochemical system operating in far-from-equilibrium conditions. Whether we understand the physics of the nonequilibrium state as well as we understand classical mechanics and equilibrium thermodynamics is another question. We do not, at the moment. But then, insisting on interpreting everything indiscriminately in the terms and concepts that we understand best and believe in, rather than in the terms and concepts that are consistent with experimental reality is not science, but a system of unsubstantiated beliefs analogous to religion. If classical mechanics and equilibrium thermodynamics work so well for non-living matter, it does not necessarily mean that they should work equally well for living matter. Common sense would actually suggest that the very fact that classical mechanics and equilibrium thermodynamics work so well for non-living matter means that they are highly unlikely to be adequate frameworks for interpretation of living phenomena, for there is a *qualitative *difference between living and non-living matter.

Last but not least, if a new theory/paradigm matches and organizes the whole of observable and measurable reality in a more elegant, simple, and intuitively clear way and is more useful in *practical terms *for understanding and prediction than the old one, why not use it? Let us, therefore, consider a few more examples of how the new image of biological organization helps with understanding and predictions.

## Flow rates versus concentrations

Equilibrium thermodynamics necessarily pays special attention to concentrations, as concentration differences near equilibrium define all movement and the direction and range of change in the world of equilibrium thermodynamics. And that is what biologists usually measure and assume to be most important. Meanwhile, one of the critical parameters characterizing the structure and dynamics of nonequilibrium systems is not concentration but *the rate of flow*. As a biologically relevant example, consider the concentration of glucose in systemic circulation of human organism. The steady-state level of glucose in the blood is maintained within a remarkably narrow concentration range, even soon after a prodigious meal or during endurance exercise. The parameter reflecting physiological state of the organism is not glucose concentration but the rate of glucose flow/circulation. The same is true for oxygen, phosphate, iron, calcium, and many other metabolites circulating with the blood flow. Symmetrically, at the sub-cellular scale, the measurements performed on over 60 different metabolites in different metabolic pathways show that intracellular metabolite concentrations are homeostatic and do not change significantly upon transitions in the physiological state of the cell, such as, for example, a shift from resting state to a high workload state, while metabolic fluxes through corresponding pathways change dramatically upon such transitions [118]. In other words, experimental reality in biology agrees with nonequilibrium thermodynamics in that the relevant parameters accurately reflecting/predicting the state of a biological system on any scale are not concentrations but flow rates.

Next, because transitions between different physiological states of a cell (or an organism) are nothing else but manifestations of organizational transitions within the complex structure of conjugated fluxes and interdependent gradients that is the cell (or the organism), other practically relevant parameters are the threshold values of individual flow rates at which organizational state transitions are triggered within a given structure of conjugated fluxes. Given that in nonequilibrium systems different fluxes differ in their relative influence on the overall structure of conjugated fluxes and gradients, i.e. some are more important/critical than others, the questions relevant for understanding physiology in normalcy and disease, from the point of view of nonequilibrium thermodynamics, are as follows: i) what are the relationships between different fluxes and gradients in a "healthy" (balanced) state of biological system, and how does the organization of the pathological state differ from the organization of the healthy state; ii) what can cause misbalances in a "healthy" structure of fluxes, leading to transitions from healthy organizational states to pathological organizational states; iii) what are the main determinants of stability for a given organizational state; iv) how can a balanced structure of fluxes be restored; and other questions of the same type. Notice that, ironically, and hardly coincidentally, nonequilibrium thermodynamics of the West is in remarkable harmony with the traditional Eastern views on the organism and on life in general, which are based on such concepts as conflict of opposites (countervailing gradients), energy fluxes, and the disease state as a misbalance of energy flow, but not with the Western conceptualization of biology and life. Locked in the box of classical mechanics and equilibrium thermodynamics, the Western biomedical sciences are doomed to interpret the diseased organism as a malfunctioning machine and, as a consequence, are exclusively preoccupied with reverse engineering of biological systems in futile efforts to infer pre-defined designs and searching for broken parts to be replaced. This may explain the jarring contrast between the plethora of resources being poured into biomedical research and the paucity of practical cures that have emerged as a result of this investment [119].

## Resolving controversies and puzzles: ion partitioning and permeability transitions

Any science has its skeletons accumulating in the form of paradoxes, inconsistencies, and contradictions, which it hides away in the closets of neglect. Of all experimental sciences, molecular and cell biology has accumulated perhaps the largest and most diverse collection of paradoxes, contradictions, and inconsistencies over many years of research. Let us pull out a couple of old skeletons from the closets of biology and take a closer look at them in light of the new conceptualization.

As an example, consider the half-century-old and bitter dispute over physical causes behind the partitioning of ions in the cell. Generally speaking, there are two main conflicting schools of thought. One can be found in all conventional biochemistry courses and textbooks. It posits that the gradients of ions across semi-permeable cellular membranes are created and maintained by continuous pumping of ions against their concentration gradients. The pumping is performed by a variety of protein pumps fueled by ATP hydrolysis, while the influx of ions occurs down their respective concentration gradients across cellular membranes through diverse ion channels, in a regulated manner. Superficially convincing and, more importantly, intuitively appealing for the mechanistic mindset, this image is not consistent with a great deal of experimental observations and has even been argued to blatantly contradict such basic physical laws as the law of energy conservation [120-122]. In fact, on a more general level, the conventional image of molecular partitioning inside the cell manifestly fails to explain a veritable museum of mouth-opening paradoxes (reviewed in [120]). As an example, consider cells with permeabilized plasma membranes that i) remain viable and functionally active, ii) do not significantly lose their contents over extended periods of time, and iii) remain visually intact on electron micrographs, while at the same time allowing the apparently unhampered diffusion of molecules as large as 800 kDa in and out of cells [120,123].

**Figure 4:**
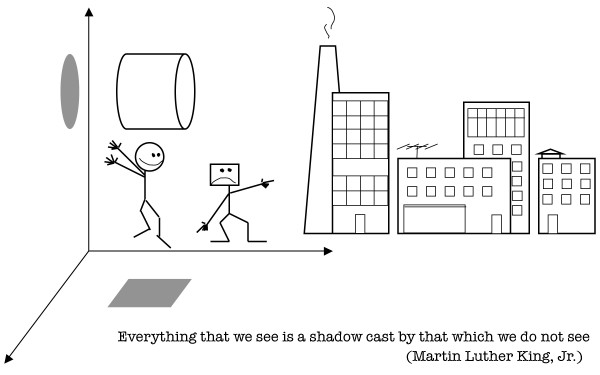
**Progress through conflict**. Restricted to two-dimensional interpretations by their shared paradigm of reality, round- and square-headed people argue whether an observed aspect of reality is a "circle" or a "square". Although both opposing views are correct, the controversy cannot be resolved without transcending the two-dimensional paradigm and re-conceptualizing reality as being three-dimensional. Because most of the objects in the two-dimensional world of the opponents have square angles, the interpretations of square-headed people are intuitively appealing, seem more believable, and, thus, will be preferred. As a consequence, square-headed people will move up the career ladder and grow in numbers much faster than round-heads. Inevitably, due to the economic principle of least effort, round-heads and their interpretations will be neglected and suppressed, as ignorance and suppression seem to cost less than the efforts of reconciling the seemingly irreconcilable. The ensuing misbalance, manifested as the absence of conflict and widespread complaisance with established order, leads eventually to the belief that reality is what it is known to be by everyone, namely a "square". Books titled "The End of Science" are published and become bestsellers [125]. Such a misbalance blocks the development of collective intelligence, which, by its nature, always proceeds through recurrent conflicts of alternatives/opposites and their constructive resolutions on increasingly higher planes of understanding. No conflict means no resolution. No resolution means no development. No development means stagnation, disease, and degradation. "Not knowing is true knowledge. Presuming to know is a disease. First realize that you are sick; Then you can move towards health." (Lao-Tzu, 600 BC) [126].

The opposing school of thought interprets the cell as a complex and dynamic mosaic of co-existing phases, in which ions (and other molecules) partition between different phases in accord with the laws of equilibrium thermodynamics without any pumping [120,122]. Needless to say, the latter interpretation is open to all sorts of critiques as well and is not consistent with a variety of well-established experimental facts–even though, in some respects, it comes much closer to the truth than the conventional interpretation. Besides, it is completely non-intuitive for the average biologist and has no appeal whatsoever for the mechanistic mindset. This alone may explain why the work of its authors and the authors themselves have been largely–and, one should say, undeservingly and regrettably–neglected. Meanwhile, as is so often the case in the history of ideas, both conflicting schools of thought are both right and wrong, depending on the aspect one chooses to consider (Fig. 4). The problem is that the experimental observations pertaining to ion (molecular) partitioning simply cannot be reconciled in their entirety in a self-consistent manner without transcending the conceptual frameworks of equilibrium thermodynamics and classical mechanics. The studies of far-from-equilibrium chemical systems, such as the BZ reaction and others, show that the emergence and maintenance of concentration gradients in nonequilibrium systems require neither membranes nor pumps (which does not mean that the effects of certain gradients and fluxes cannot be superficially reminiscent of the effects expected from membranes and pumps). It is thus reasonable to suggest that the gradients of ions observed in the cell are different from the familiar gradients of equilibrium thermodynamics in the sense that they represent nonequilibrium *steady state fluxes *of ions dynamically partitioned in space and time. In other words, the majority of ions involved in maintenance and functioning of the living state exists not as free-diffusing ions (most of the time), but as moving ions in the form of ion fluxes microcirculating on multiple spatiotemporal scales around, along, or within cytoskeletal structures, cellular membranes, and other sub-cellular structures and multiprotein complexes where relatively high concentrations of ions are usually observed, such as, for example, endoplasmic reticulum, mitochondria, and the A-band in striated muscle cells. Notice that, superficially, localized circulation of ions within a multiprotein complex/structure/organelle may appear either as an ion "store" (by necessity requiring membranes, pumps, channels, and other "machinery") or, alternatively, as absorption of ions on proteins (phase partitioning), and it would inevitably (and mistakenly) be interpreted in such ways within the frameworks of equilibrium thermodynamics and classical mechanics. Nonequilibrium thermodynamics, on the other hand, would infer from the same set of data that there exists a conjugated flux or fluxes that fuel local (global) circulation of ions. What are the conjugated fluxes/gradients that drive the circulation of ions remains to be determined. One of them may well be the flux/circulation of phosphoryl driven by coupled phosphotransfer reactions [99,124]. Notice that such an interpretation, while being consistent with the majority of, and perhaps all, well-established experimental observations, readily reconciles the arguments and counter-arguments put forward by both the proponents of pumps and the advocates of phases. It also helps to understand why contents do not leak from cells with permeabilized plasma membranes, unless certain structures (fluxes, in fact), such as actin filaments, for example, are not destroyed by drugs [123]. Being a system of intertwined molecular fluxes and coupled gradients, with ions, small molecules, proteins, lipids, and other macromolecules forming a convection-like, multi-scale spatiotemporal pattern manifested as cytoarchitecture, the cell can apparently preserve most of its structures and functions for extended periods of times, even when its plasma membrane is severely compromised. This implies that the plasma membrane is not a conventional membrane of the mechanistic world, but is a part of a convection-like pattern of energy/matter exchanges that is relatively dispensable for the organization and dynamics of the remaining part of the pattern in a short run. This also implies that actin filaments themselves and/or the fluxes of energy/matter intimately associated with the cytoskeleton represent the keystone fluxes that hold a convection-like organization of intracellular architecture together.

One of the logical inferences that can be immediately made from the above image is that the mitochondrial permeability transition (MPT), which plays a key role in the process of cell death, is caused not by the opening of some undefined and mysterious membrane pore(s) [127], but because of the weakening of one of the keystone molecular fluxes that sustain the mitochondrion as an organized, steady state, convection-like process. A decrease in the flow rates of certain key molecules through the mitochondrial structure beyond critical values would cause a stepwise hierarchical relaxation of the system of conjugated fluxes and gradients that are the mitochondrion, leading, at first, to its reshaping and restructuring and, in the end, to the loss of overall mitochondrial structure and release of mitochondrial contents into the solution phase of the cytoplasm. In other words, conceptually, the mitochondrion "dies" like an eddy and not like a punctured balloon. It is not surprising then that the physical correlate of the MPT pore has remained elusive for such a long time despite intense research efforts of defining it. The same is likely to be true for all other notoriously elusive "machinery", such as, for example, the one that mediates store-operated Ca^2+ ^entry, or the one that is responsible for Ca^2+ ^leak from intracellular stores [128,129], among many others.

Notice that the interpretation of the cell (and any living organization) in terms of nonequilibrium thermodynamics implies that fluxes, their rates and their interrelationships play the primary and defining roles in the organization and behavior of the cell (and of any living organization), whereas the interpretational framework of equilibrium thermodynamics assumes that the key parameters governing cellular organization and dynamics are concentrations and concentration gradients near equilibrium. Since a nonequilibrium system of conjugated fluxes presupposes structured circulation, organizational dynamics, and continuous change, the framework of nonequilibrium thermodynamics necessarily presupposes a fundamental role of organizational structure and circulation in the life of the cell (and any living organization). Naturally, mainstream biological research conducted within the interpretational framework of classical physics has a strong vested interest in denying the existence of intracellular organization and circulation or, where and when such a denial becomes unfeasible due to stubborn experimental facts, in downplaying their significance, for the failure to deny, to downplay, and to suppress the all-important role of intracellular organization and circulation in the physiology of the cell would immediately send to the museum of irrelevant ideas a very large fraction of the interpretations, conclusions, and promises being published and promoted in leading biological (and other) journals.

## On intracellular structure and circulation

In reality, the existence of the elaborate and continuous macromolecular structure that fills the interior of the cell is not any news for any curious cell biologist or physiologist who has worked in the field long or hard enough. There is plenty of experimental evidence that either explicitly shows (microscopy) or unambiguously implies (biochemistry) the existence of intracellular structure and circulation. The following reviews and research papers may serve as nodes of entry into a large but sparse network of inter-referenced literature on intracellular structure and circulation: microscopic visualization of intracellular structure [130-132]; intracellular circulation and metabolism [118]; extension of Coulson's flow theory of metabolism [133] to the cell interior [93,94,134]; and various biochemical studies on intracellular organization [80,81,95,123].

What follows next is a conceptual image of intracellular organization culled from multiple publications on the subject. It is a generalized image, which is meant only to convey universal features and principles of intracellular organization and dynamics. It does not describe any concrete cell. Nor does it claim to be accurate in details. The particular is specific and varied. The universal is general and invariant.

**Figure 5:**
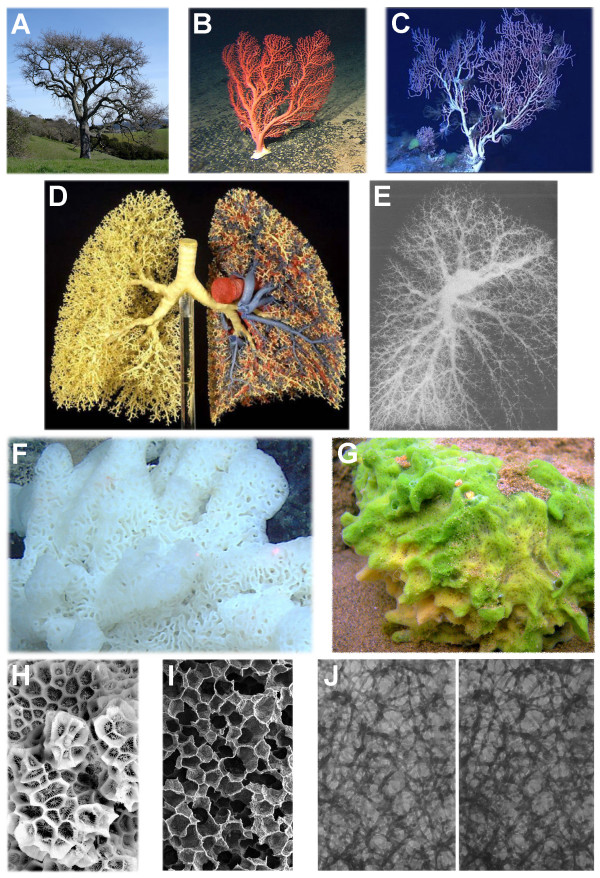
**The "trees" and "sponges" theme in biological organization**. **A**) A California oak tree; **B, C**) *Paragorgia *corals (coral images courtesy of the National Oceanic and Atmospheric Administration (NOAA) [158]); **D**) Bronchial tree of the human lung (left half of the cast) and the airways with the pulmonary arteries and veins (right half of the cast) (image courtesy of the author of the cast, Dr. Walter Weber, Institute of Anatomy, University of Bern); **E**) Arterial system of the human lung (image courtesy of Julius H. Comroe, Jr. [159]); **F**) White sea sponge (image courtesy of NOAA [160]); **G**) The freshwater sponge, *Spongilla lacustris *(image courtesy of Kirt L. Onthank, Washington State University, WA); **H**) Coral *Favites abdita *(close up) (kindly provided by Hunterian Museum, ^©^Hunterian Museum and Art Gallery, University of Glasgow); **I**) Scanning electron micrograph of alveolar tissue (image courtesy of Prof. Peter Gehr, Institute of Anatomy, University of Bern); **J**) Stereo pair of high-voltage electron micrographs showing the structure of the cytomatrix in a cultured NRK cell after rapid freezing (-185°C) and drying from the frozen state (-95°C) (reproduced with permission, ^©^Porter, 1984. Originally published in The Journal of Cell Biology. 99: 3s–12s, [130]).

It is useful to imagine the cell interior as a sponge-like structure, in which a solution phase fills and circulates through the channels and pores of various sizes perforating a dynamic soft-matter phase made of diverse steady-state macromolecular structures, compartments, and organelles (Fig. 5). The constituent parts of the soft-matter phase are dynamically coupled to each other through direct transient physical associations and/or, indirectly, through the circulating solution phase. Like cardiovascular or pulmonary systems of animals, the cellular sponge is dynamic and plastic, with different parts of the sponge changing and rearranging on different spatiotemporal scales. Some parts of the intracellular soft-matter phase change slowly, giving the impression of stable architectures. Other parts display an almost water-like fluidity. Yet other constituents show intermediate dynamics. In other words, the structure and dynamics of intracellular organization cover a wide range of spatiotemporal scales. On the whole, of course, the intracellular circulation system operates and changes on vastly smaller spatial dimensions and faster timescales than does the organism-wide system of central circulation. However, the general features of, and physical principles behind, the organization and dynamics of both systems have been proposed to be the same [79,135].

On the basis of self-similarity of scales in biological organization and dynamics [79] and biochemical evidence [93,94,118], one can envisage, for example, that the "macro"-channels perforating the soft-matter phase branch directly into the physical tunnels mediating substrate channeling within multiprotein compartments, complexes, and individual enzymes, thereby forming a continuum of intracellular circulation in the form of a tree-like hierarchical system of branching dynamic channels. In this case, the multiprotein complexes associated with membranes and cytoskeletal structures would function as dynamic walls of dynamic "veins" and "arteries" of the intracellular circulation system, while substrate channels of multienzyme complexes and compartments [80,81] and intramolecular tunnels of enzymes [84,85] would perform as molecular "arterioles".

Since intracellular circulation is intimately coupled to central circulation [94,118], the organism as a whole represents and performs as a multi-scale continuum of circulation, which includes central circulation, microcirculation in tissues, intracellular circulation, circulation of metabolites within channels/tunnels of multienzyme complexes and individual enzymes, and down to the scale of elementary particles circulating within individual protein structures. Indeed, substrate channels of multienzyme complexes and intramolecular tunnels of enzymes branch into multiple individual active sites where chemical transformations take place. In turn, individual active sites of enzymes are lined up by the amino acids belonging to the evolutionary conserved networks of physically interconnected and thermodynamically linked amino acids that mediate protein-wide distribution/transport of energy/matter within individual protein structures and that perform, essentially, as intramolecular "capillaries" [39,136-140]. Beating hearts of living creatures may thus literally pump the circulation of elementary particles within the living matter! The reverse may also be true: a sudden entanglement of elementary particles may prompt individual hearts to start beating in unison. In other words, "miracles" become possible once an inadequate conceptualization is replaced by the adequate one, for it is not the reality that sets our limits but our interpretations of reality.

One of the *practically *useful inferences that can be immediately made from the image outlined above (and see more below) is that the protein structure represents and performs, in fact, as a hierarchical system of branching submolecular pathways/channels that mediate flow/circulation, exchange, and transport of energy/matter on the molecular and atomic scales, and below. In other words, in the same way as the cardiovascular system functions as an interface coupling and integrating the circulation of energy/matter within and across cellular and organismal organizational levels, the protein structure functions as a dynamic interface coupling and integrating the circulation of energy/matter within and across subatomic, atomic, and (macro)molecular levels of organization.

Pertinently, the idea of organized electron flow through proteins, protein complexes, and intracellular organization as a whole was suggested as early as 1941, by Albert Szent-Gyorgyi, a famous Hungarian biochemist, the discoverer of vitamin C, and a Nobel laureate, who also felt that the cell represents and functions as an energy continuum [141]. Although, at the time, the idea of electron conduction in proteins was rejected by physicists on theoretical grounds (like many other *practically *useful physical phenomena, such as high-temperature superconductivity, for example), the experimental demonstration of electron and proton tunneling in proteins [142-144] led later to the revival of interest in Szent-Gyorgyi's ideas. Currently, long-range electron and proton transfer in proteins (ET and PT, respectively) as well as the intimate connections among ET/PT, enzymatic catalysis, and protein structure and dynamics is the subject of intense research efforts, promising a drastic re-evaluation of classical models of enzymatic catalysis [39,145-148].

It is worth emphasizing the symmetry and coupling of scales within the organization of organism-wide circulation. The central circulation system of an organism is made up of macroscopic arteries, arterioles, and capillaries. It functions as a physical transport system mediating organism-wide distribution and exchanges of energy/matter/information. In a mature organism, it is dynamically maintained as a tree-like system of the hierarchically branching channels made of coupled specialized cells and transporting anything from protons, ions, water, small molecules, macromolecules to large macromolecular complexes, particles, and whole cells. The physicochemical composition of blood is very complex and is subject to homeostatic regulation. The postulated "arteries", "arterioles", and "capillaries" of the cell interior represent a physical transport system that mediates cell-wide distribution and exchanges of energy, matter, and information. This system is made up of coupled macromolecular complexes/compartments and transports anything from protons, ions, water, small molecules, and macromolecules to large macromolecular complexes and organelles. The chemical composition of cellular "blood" is very complex and is subject to homeostatic regulation [118]. The rates of intracellular circulation and central circulation are known to correlate with each other, implying coupling and interdependence of scales [94,118]. Increased rates of blood circulation, for example, create permissive conditions for higher metabolic rates at the cellular level and are generally accompanied by accelerated intracellular circulation and increased metabolic rates within working tissues and cells [93,94,118]. The reverse is also true. Increased rates of intracellular circulation in specific tissues–consider, for example, the hormone-stimulated "fight-or-flight" response–creates permissive conditions for achieving a high metabolic output at the organismal level and is normally accompanied by increased rates of both central circulation and local microcirculation in the target tissues.

Taking into account the symmetry of scales not only in space but also in time, one discovers that one of the best descriptions of the basic features and principles of intracellular organization and dynamics can be found in invertebrate zoology textbooks, in the chapters describing the phylum *Porifera*, i.e. sponges. The generalized sponge is an almost perfect organizational replica of the generalized cell. The sponges are aquatic animals leading a sessile filter-feeding lifestyle by continuously circulating water through their bodies. Structurally, a living sponge represents a multi-chamber, porous, jelly-like endoskeleton, which is made, populated, and continuously remodeled by a colony of mobile specialized cells that are organized and perform in essentially the same way as specialized proteins are organized and perform within the cell [149-151] (Fig. 5). It is worth noting that, while lacking any kind of nervous tissue, a colony of cells comprising a sponge normally behaves as a highly coordinated whole. However, the mechanisms of such coordination are unknown. According to the principle of self-similarity of scales within the living matter continuum [79], the remarkable organizational parallels between the cell and the sponge are hardly coincidental, and may suggest that i) the first cells emerged, in fact, as sessile filter-feeding colonies of proteins and other macromolecules organized around an endoskeleton made, populated, and continuously remodeled by proteins and other molecules in accord with changing environmental conditions and/or (economic) needs of a given colony; and ii) the essentials of both the primordial microenvironment and the organizational dynamics of ancient macromolecular colonies are, in fact, preserved inside modern cells, i. e. the detailed history of primordial evolution is "remembered" by Nature and can be directly "read" by analyzing intracellular organization and dynamics. Pertinently, fossils of all existing types of sponges have been found in rocks that are more than half-a-billion years old, suggesting that the sponges represent one of the most ancient and persistent organizational forms of life [151].

Concrete physical realizations of the general principles of cellular organization outlined above can be very diverse, as exemplified by a variety of specialized/differentiated cells within a multicellular organism. Notice that, consistent with the postulated self-similarity of scales within the living matter continuum [79], the physical appearance, organization, and dynamics of specialized cells in multicellular organisms often, if not always, resemble, in some way or another, the organization and dynamics of their immediate environments, i.e. the larger-scale structures/tissue that these cells form. Skeletal muscle cells, for example, resemble skeletal muscle fibers in their overall organization, appearance, and dynamics. Secretory cells resemble their native secretory glands/tissues. The organization and dynamics of neurons, the cells that integrate and process multiple inputs and multiple spatiotemporal scales within their bodies, are reminiscent in their function and overall organization of the brain integrating and processing multiple inputs and multiple spatiotemporal scales within its tissue.

While discussing universal features of intracellular organization, it is useful to consider at some length a recent intellectual breakthrough in our understanding of certain phenotypic universalities in biology, namely, the general physical model put forward recently by West, Brown, and Enquist to explain allometric scaling laws [152].

It was noticed early on by biologists that, despite a bewildering diversity of sizes and appearances of life forms, many parameters (*Y*) pertaining to basic biological processes relate to the organism mass (*M*) in a simple and universal fashion, namely, *Y *~*M*^*b*^, where the power exponent (*b*) takes values that are multiples of 1/4. The relations between organismal mass and various biological parameters are known in biology as allometric scaling laws. The best-known of these laws was introduced by Kleiber, who noticed that basal metabolic rate in mammals and birds scales as the 3/4 power of body mass [153]. Subsequent research confirmed his observations and showed that Kleiber's law holds for nearly all biological organisms, including animals, plants, and unicellular organisms [154]. It was also found that lifespan scales as *M*^1/4^, heart rate scales as *M*^-1/4^, lengths of aortas and heights of trees scale as *M*^1/4^, while radii of aortas and tree trunks scale as *M*^3/8^, and so forth [154]. For a long time, however, these empirical biological laws have remained without theoretical foundation, i.e. unexplained.

Approximately a decade ago, West, Brown, and Enquist (WBE) proposed a theoretical model that elegantly rationalized biological scaling laws and quantitatively predicted a variety of phenotypic parameters for a remarkably large range of biological phenomena and systems [152,154]. Briefly, the authors proposed that, since any biological organism is made of small and multiple constituent parts that have to be supplied by metabolites and relieved from waste products, and since any biological organism evolves under competitive pressures to maximize its metabolic power, while minimizing resource expenditure, natural selection led to the emergence of a universal physical design for resource distribution/transport systems within organisms, a fractal-like hierarchical tree of branching pipes, as exemplified by the physical organization of the bronchial tree and of the cardiovascular, nervous, and other systems [154,155] (Fig. 5). Because fractal-like geometry maximizes the surface area of exchange, while minimizing resource expenditures for its maintenance and function, the universal features of biological trees, such as hierarchical branching and fractal-like organization, are omnipresent in Nature, as they are enforced by economics and evolutionary competition. Pertinently, the ideas of universality in biological tree design and its relation to economy can be traced back in time to Leonardo da Vinci, who noticed and documented area-preserving branching of biological trees [154,156], and to the father of fractals, Benoit Mandelbrot, who titled his principle work "The Fractal Geometry of Nature" [155].

The WBE model is based on a number of explicit and implicit postulates: i) resource distribution trees are space-filling, as they are meant to service all metabolically active constituent parts; ii) the terminal units of branching trees are invariants; iii) performance of the resource distribution trees is maximized by minimizing the energy and other quantities required for resource distribution; iv) metabolic rates are constrained by the rate of resource supply; v) natural selection enforces maximization of metabolic output and metabolic efficiency; and other postulates [135,152,154,157]. In their later publications, by relaxing certain assumptions, the authors generalized and extended the WBE model up and down the scale, from individual molecules to ecosystems [135,154]. Unfortunately, being apparently (and unsurprisingly) unfamiliar with the large but sparse body of experimental work on intracellular organization and circulation, which, by necessity, is condemned to an underground existence in the mainstream research literature, the authors had to resort essentially to "hand-waving", while extending their model to intracellular organization: "The observation that *b *= 3/4 for unicellular as well as multicellular organisms suggests that the distribution networks within single cells obey the same design principles. ... As we shall show, the success of extending allometric scaling models down to the molecular level raises the question of whether there is a real or "virtual" hierarchical transport system inside cells. ...In any case, the data and their theoretical underpinnings define the problem and suggest that systems that supply cellular metabolism must have fractal-like properties." [135]. It is worth noting that this rather sad fact illustrates once again that the conventional interpretational framework of classical physics does not simply function as a source of convenient and appealing metaphors and approximations in biology, but for all *practical *matters, acts as a damaging and misleading ideology that justifies systematic suppression of relevant and useful ideas and experimental facts, while promoting and rewarding their opposites.

Placing the work of West, Brown, Enquist and their colleagues and exponents in the context of our discussion, one can now properly appreciate the power of the WBE model, which not only implies the existence of an intracellular circulation system, but accurately predicts its physical form, i.e. a hierarchical system of branching channels mediating distribution and exchange of energy/matter within the cell. It should be pointed out that fractal-like trees become space-filling, turning effectively into sponges, once the "colonization" of three-dimensional space becomes sufficiently dense (consider, for example, leaves of botanic trees, alveoli of bronchial trees, and cells in organisms). All living biological trees/sponges are dynamic, with smaller branches changing faster than larger branches. Overall, the organizational dynamics of a given biological tree/sponge normally covers a large range of spatiotemporal scales. At the same time, the organizational dynamics of different classes of biological trees/sponges cover different but overlapping ranges of spatiotemporal scales. Because the major part of intracellular organizational dynamics takes place on extremely short distances and fast timescales, the architecture of intracellular tree/sponge has been difficult to capture and visualize. Historically, high-voltage electron microscopy was the first method that revealed a fine sponge-like organization of the cytoplasmic matrix (Fig. 5J) [130]. Later on, the introduction of ultrathin, resinless sections allowed for visualization of the cytoplasmic matrix with conventional electron microscopes [131]. As shown in Figure 5, the physical appearance of the cytomatrix on electron micrographs is consistent with the general features and principles of intracellular organization that can be inferred from the symmetry of scales in biological organization and dynamics (compare H, I, and J in Fig. 5).

Notice also that, according to the WBE model, metabolic output, metabolic efficiency, and evolutionary competition play the fundamental and defining roles in shaping physical organization of biological organisms. It is not difficult to see that metabolic output, metabolic efficiency, and evolutionary competition are biological equivalents of the more general concepts–economic production, economic efficiency, and economic competition–which, as suggested herein and elsewhere [79], govern the spatiotemporal organization and dynamics of all biological organizations comprising the continuum of living matter, from macromolecules, cells, and organisms to organizations, economies, ecosystems, and the whole planet as an integrated system of life.

Similar to so many other long-lasting controversies in biology, which persist unresolved simply because arguments and counter-arguments are irreconcilable within the frameworks of classical physics, the controversy surrounding the WBE model/theory cannot be resolved without transcending the mechanistic framework where the model was born and remains. In this regard, it should be pointed out that, in reality, the internal resource distribution/transport systems of biological organisms (at all scales) are not mechanistic pipes built according to a pre-conceived design, but dynamic and adaptive fluxes of energy/matter in themselves, shaped by both internal and external influences. And their main purpose is not to deliver resources and remove waste–that is the limited interpretation of the mechanistic paradigm–but to integrate energy/matter and space into one scale-free continuum of energy/matter circulation. In other words, it is not that "the geometry of the vascular network governs how a suite of organismal traits covary with each other, and, ultimately, how they scale with organism size" [161], but rather it is that, because the scales within the continuum of living matter continuously strive to become self-similar and to co-vary under the pressure of economic competition, the interface integrating the energy/matter/information exchanges across and within multiple scales within a given biological organism necessarily follows fractal-like organization. This means, by the way, that the persistent disruption of fractality and/or relative decline in fractal complexity in biological organization and dynamics signify uncoupling of scales, a loss of covariation and interdependencies across scales, and, as a consequence, a decline in functional performance of a multiscale whole, which are all typical manifestations of disease and aging, as documented in a variety of physiological and morphological studies performed at different levels of biological organizational hierarchy [162-167]. Pertinently, the modern spectroscopy and imaging technologies, which are being developed for noninvasive, rapid, and accurate detection of precancerous and cancerous lesions on the basis of structural changes taking place in diseased cells and tissues, readily reveal both the fractal organization of intracellular structures and the deterioration of long-range correlations within diseased cells and tissues [165,168].

## Summary, conclusion, and ramifications

Whether explicitly stated or tacitly implied, the phenomena studied in molecular and cell biology are traditionally interpreted and rationalized within the conceptual frameworks of classical mechanics and equilibrium thermodynamics. Accordingly, the conventional image of the cell carries within it all the familiar logic, inferences, and assumptions of classical physics. Simplifying, the cell exists and functions because a genetic program encoded in the DNA directs the expression of a specific set of proteins and RNAs that have certain structures, activities, and functions, as specified by evolutionary design. Having predefined structures, activities, and functions, proteins and RNAs assemble themselves in a predetermined way into macromolecular complexes, machines, and larger sub-cellular structures to perform evolutionary predetermined functions, and to achieve specific ends in accordance with currently active cellular programs, such as cell proliferation, cell division, cell differentiation, apoptosis, endocytosis, and others. Naturally, such a mechanistic/clockwork image of the cell has high legitimacy and appeal because it is so consistent and harmonious with our everyday experiences in the world of the classico-mechanistic reality, which is socially constructed [169] and maintained in industrial societies by education, socialization, and living experience. Notably, the mechanistic/clockwork image of the cell justifies and endorses extreme attitudes in reductionism and specialization, for the belief is that, whatever its complexity may be, the evolutionary design of the cell is there and we will sort it out sooner or later, in all its details and intricacies, by means of reductionism, specialization, reverse engineering, and the crude force of ever-advancing research technology and methods. The problem is that, instead of clarifying the hypothetical evolutionary design, ever-advancing technology and methods make it ever more confusing and elusive, by generating massive amounts of the experimental data that is manifestly inconsistent with and difficult or impossible to assimilate within the mechanistic/clockwork image of the cell [12-17]. Hence, the accumulation of paradoxes, controversies, inconsistencies, and contradictions that persist unresolved over time; hence the rise of technology-driven "discovery" science and a decline of hypothesis-driven research – a sure sign of the failure of the conventional paradigm to serve as a theoretical framework enabling understanding and prediction of experimental outcomes.

The present work shows that the experimental reality in molecular and cell biology becomes largely devoid of paradoxes, inconsistencies, and contradictions, and is thus best understood, if the conventional interpretational framework of classical physics is replaced by an alternative paradigm of biological organization, which is based on the concepts and empirical laws of nonequilibrium thermodynamics. In addition to resolving paradoxes and controversies, such a paradigm shift reveals hitherto unappreciated connections among many seemingly unrelated phenomena and observations, and provides new and powerful insights into the universal principles that govern the emergence and organizational dynamics of living systems on each and every scale of biological organizational hierarchy, from proteins and cells to economies and ecologies [79].

In these concluding remarks, let us summarize main concepts and assumptions of the new paradigm of biological organization and comparatively evaluate the practical utilities of the old and new paradigms as theoretical frameworks enabling understanding and prediction of experimental reality.

Studies performed on relatively simple open inorganic systems of interacting molecules show that spontaneous dynamic compartmentalization in space and time, cooperative behaviors, and macroorganization invariably take place within an open physicochemical system of interacting microcomponents, provided the system is driven far away from equilibrium by continuous flow of energy/matter passing through the system. Although the precise physical principles and forces behind such self-organizational phenomena are not clear at present, certain empirical law-like organizational patterns discovered in studies on open nonequilibrium inorganic systems can explain and predict organizational dynamics of many, and perhaps all, far-from-equilibrium systems, including living organizations.

The pertinent self-organizational patterns discovered in nonequilibrium thermodynamics are as follows (see Fig. 3). The macrostructures spontaneously emerging in open nonequilibrium systems of interacting microcomponents are of a steady-state nature and exist as metastable configurations of conjugated fluxes and interdependent gradients or, in other words, as metastable flow/circulation patterns structured on multiple scales of space and time. Increasing the rate of energy/matter flow through an open nonequilibrium organization/system of interacting components leads to the growth of the organization/system in size and complexity. The increase in complexity proceeds through stepwise organizational transitions from states of relatively low order (low negative entropy) to states of relatively high order (high negative entropy) and is accompanied by the formation of multi-scale organizational hierarchies. Maintaining a nonequilibrium organization/system at a given level of order and complexity requires a continuous and stable flux of energy/matter through the system. Decreasing the rate of energy/matter flow through an organization/system leads to a stepwise hierarchical relaxation of its organizational structure and a loss of complexity and order, culminating ultimately in the dissolution and death of the organization.

Since few would argue that the cell is not an open nonequilibrium physicochemical system of interacting components or that cellular organization and dynamics do not obey physics, it is just natural to assume that the cell or any functional constituent of the cell exists and performs as a multi-scale dynamic organization of conjugated fluxes and interdependent gradients, i.e., a dynamic structured pattern of energy/matter flows, which obeys empirical laws of nonequilibrium thermodynamics. Importantly, this implies that molecular macroorganization, compartmentalization, and structuring within the cell are not pre-determined by some pre-existing evolutionary design, but are driven by the same physical principles and forces that drive self-organization in open, inorganic, far-from-equilibrium systems studied in the field of nonequilibrium thermodynamics. As suggested in this work, the principle difference between inorganic and living organizational processes is that functional constituents of living systems (on any scale) are complex living organizations in themselves, whose structure and dynamics have been shaped/biased (but not deterministically specified!) by evolution. The structures and dynamics of all living organizations, from proteins and cells to societies and ecologies, embody their evolutionary histories/memories. Therefore, in contrast to inorganic systems, the self-organization of any biological organization, such as the cell for example, is greatly facilitated, and to a certain degree governed (but not determined!), by evolutionary memories embodied in specific, but flexible and adaptive, structures and dynamics of its constituents.

It is worth pointing out that the cell as a whole or any functional constituent of the cell is dynamic in two different senses. The cell or a functional part of the cell is dynamic in the sense that it represents a structured pattern of continuous energy/matter flow. At the same time, the cell or any functional part of the cell is dynamic in the sense that this structured flow pattern can adopt several, and potentially many, metastable organizational configurations that differ in the organization of energy/matter exchanges transiently maintained among the interacting components that make up (and flow through) the pattern. The latter type of dynamics, which may be called configurational dynamics, as opposed to flow dynamics, requires and relies on flexibility and adaptability of functional constituents comprising a given biological organization. This, in turn, implies that configurational plasticity and adaptability should be necessarily enforced and preserved by evolution on each and every level of biological organizational hierarchy. Finally, it is important to emphasize that the re-interpretation of the cell and biological organization in terms of nonequilibrium thermodynamics implies that the critical parameters defining the organization and dynamics of living systems are flow rates and not concentrations, as tacitly implied in conventional interpretations rooted in the framework of equilibrium thermodynamics.

The cell as a molecular system should be then pictured as a multi-scale arrangement of metastable molecular flow/circulation patterns. The flow/circulation patterns comprising the cell are interdependent and mutually morphing, being integrated into one whole of the cell by fluxes of shared components. Metastable flow/circulation patterns are physically manifested as various steady-state sub-cellular structures, compartments, and macromolecular complexes that make up the cell. Various metastable flow/circulation patterns continuously compete and cooperate one with another in order to obtain and to ensure stable and accelerating flows of energy/matter passing through them, which they require for their maintenance and growth within the cellular economy they comprise. In exactly the same way, various business, social, and political organizations compete and cooperate one with another in order to obtain and to ensure stable and accelerating flows of energy/matter passing through them, which they require for their maintenance and growth within the socio-politico-economic system they form. Those organizations that succeed in securing and accelerating the flow of energy/matter through their structures grow in size, order, complexity, and influence. Those organizations that fail to maintain achieved rates of energy/matter flow through their structures either diminish in their relative size, order, complexity, and influence or dissolve. This implies that whenever one observes the emergence and growth or persistence of biological organization/structure, one should assume the existence of an accelerating or relatively stable and rapid flux of energy/matter passing through biological organization/structure in some form. The converse is also true: behind the disorganization and dissolution of any biological organization/structure there is always a weakening of the energy/matter flux(es) sustaining the organization/structure.

As a relevant and concrete example, consider the study on dynamic compartmentalization of glycolytic enzymes mentioned earlier in our discussion. This study demonstrates that glycolytic enzymes reversibly partition from a soluble pool to a mitochondria-bound pool upon increased respiration and back into the soluble pool upon inhibition of respiration. Mitochondrially-associated enzymes form a functional glycolytic sequence that supports mitochondrial respiration through substrate channeling [98]. The new paradigm of biological organization explains this phenomenon in the following way. Respiring mitochondria create a massive demand for pyruvate. To satisfy this demand, increased production of pyruvate can potentially be achieved inside the cell in a number of different ways, for example, by boosting the expression and concentrations and/or activities of soluble glycolytic enzymes or by increasing the rate and efficiency of metabolic flux through glycolytic pathway by means of compartmentalization and coordination of glycolytic enzymes and substrate channeling. Economically speaking, the arrangement of energy/matter exchanges that ensures the fastest flux of energy/matter through the glycolytic pathway with the least expense of cellular resources will win the economic competition with alternative arrangements and eventually prevail. Physically speaking, in accordance with empirical laws of nonequilibrium thermodynamics, an accelerating flux of energy/matter through the glycolytic pathway, with mitochondria acting as physical "sinks" for pyruvate, is expected to lead to the self-organization, compartmentalization, and association of glycolytic enzymes with mitochondria, provided the rate of the flux is high enough. The self-organization, compartmentalization, and association of glycolytic enzymes with mitochondria are greatly facilitated by the evolutionary memory of such an arrangement, which is embodied in the specific structures and dynamics of glycolytic enzymes. If the rate of the energy/matter flux through the glycolytic pathway slows down beyond a certain threshold level, either because mitochondria stop respiring, or because mitochondria start using alternative sources of pyruvate, or because of other (potentially multiple) reasons, mitochondria-associated glycolytic compartments will disorganize and glycolytic enzymes will repartition into the soluble cytoplasmic pool. Notice that the described organizational dynamic is explained not in terms of some putative designs and programs, which would be *ad hoc *and ultimately unverifiable hypotheses (potentially multiple), but in terms of universal physical laws applicable to all open far-from-equilibrium systems.

A conceptually correct and revealing metaphor here is an assembly line of an automobile factory. When there is a weak demand for cars and no competition between automakers, the specialists performing individual steps of the car assembly sequence can potentially work and produce automobiles in a spatiotemporally disorganized manner, simply by randomly wandering around, encountering partially assembled cars, and performing on them their parts of the assembly work. Although possible, such a stochastic and disorganized production is slow and inefficient. There is no flux and no assembly line as such. An increasing demand for cars and escalating competition between different automakers would lead to the emergence of an assembly line and a flux of energy/matter passing through it. Such a flux would keep individual specialists organized and coordinated in space and time. If the car assembly flux is relatively slow, individual specialists may wander away for a short while without affecting the rate and efficiency of production. A relatively fast flux would naturally require a higher degree of spatiotemporal order and coordination from the workers (a state of higher negative entropy). However, the organization of an assembly line is of a steady-state nature in both cases. Each worker can be and is replaced from time to time by an analogous specialist (or even by a generalist at relatively low flux rates). Only at extremely high rates of the assembly flux, the organization of the assembly line may cease to be steady state, for there is simply not enough time to replace individual workers without disrupting the assembly process as a whole. Although high flux rates are required for and conducive to spatiotemporal self-organization, coordination, and high production efficiency, excessively fast fluxes often lead to organizational instabilities that eventually result in cascading breakdowns, disorganization, and restructuring. This may explain *pari passu *the origins and mechanisms of self-organized criticality in biological systems, a phenomenon first noticed and described by Per Bak [170]. Finally, it is worth pointing out that, evolutionary speaking, the assembly line as an organizational pattern has not been designed, but *invented, remembered, and reproduced*, undergoing, in the process of its reproduction, all sorts of improvements, modifications, and adaptations to the demands of a given time and location, and thus evolving in space and time.

It is not difficult to see that the organizational patterns outlined above accurately recapitulate and predict the organizational dynamics observed in multiple studies on dynamic metabolic compartmentalization (see examples in the "Dynamic compartmentalization and substrate channeling in cellular metabolism" section).

In fact, as a general scheme, the described organizational patterns are scale-invariant, i.e., universal. They are applicable to and help to understand and predict self-organizational dynamics in nonequilibrium systems at all scales of biological organizational hierarchy, from proteins and cells to societies and ecologies. To express them in generalized scale-invariant terms, limiting amounts of energy/matter in some form (resources/substrates) and a strong and increasing demand for energy/matter in some form (products) create a "gradient" and a potential for the emergence of intense flux of energy/matter between the "source" of resources/substrates and the "sink" for products. This "gradient" drives then the self-organization of individual agents–who continuously search for, obtain, transform, and exchange energy/matter forms–into a steady-state organization that feeds on the flow of energy/matter passing through it, consuming available resources/substrates (inflow/input of energy/matter), and exporting products or simply dumping products/waste into the environment (outflow/output of energy/matter). In this way, an organization emerges that creates, feeds on, and accelerates an energy/matter flux down the "gradient". Individual agents/constituents comprising the organization are steady-state, open, metastable, nonequilibrium organizations in themselves and thus require stable and accelerating fluxes of energy/matter passing through their individual organizations/structures in order to survive and to grow in size and complexity. There are two important differences between the organization and its constituents that are worth mentioning. First, constituents live and operate on smaller and faster scales of space and time in comparison with the spatiotemporal scale on which their organization lives and operates. Second, the energy/matter forms that are exchanged at the scale of constituents, and that mediate the structuring of the constituents involved in exchange in a given configuration, are normally different from those that are used for the same purposes at the scale of organizations. The "interests" of individual agents/constituents in obtaining, stabilizing, and accelerating energy/matter fluxes passing through their individual structures are aligned with the "interest" of the organization they form. They are best satisfied upon the success, growth, prosperity, and persistence of their organization, which lives and evolves on its own scale in conditions of eternal and continuous economic competition with alternative organizations and unattached individual agents. As far as, and as long as, the interests and activities of an organization and its constituents become and remain aligned, the organization and dynamics at the scale of the organization and at the scale of its constituents become and remain coupled. Such dynamic coupling and ensuing interdependences across scales are necessary pre-requisites for both the "health" and the competitive performance of any multi-scale biological organization. Notice that, within the new paradigm of biological organization, economics explains nonequilibrium thermodynamics and, at the same time, nonequilibrium thermodynamics explains economics, making economics and nonequilibrium thermodynamics look like two different descriptions of one and the same phenomenon. I would like to suggest here, therefore, that economics holds keys to the understanding of nonequilibrium thermodynamics, while nonequilibrium thermodynamics holds keys to the understanding of economics, and that both of them hold keys to the understanding of biology. In other words, what appears to be three disparate sciences are, in fact, intimately interrelated aspects/dimensions of one and the same science [79].

Finally, it is important to point out that classical physics and, by implication, all modern sciences studying biological (broadly defined) phenomena and systems are "flat" in the sense that they are largely unaware of and do not make use of such a "dimension" as scale and the symmetries associated with the scale dimension [171]. Any biological system, from proteins and cells to societies and ecologies, is a multi-scale organization of metastable energy/matter flow/circulation patterns, in which multiple interdependent scales contribute to and *are required *for proper functioning and competitive performance of the biological whole. The holistic nature and multi-scaleness of biological organization require and thus imply the existence of an economically efficient physical system of communication and transport that integrates energy/matter flows both within and across scales inside any given biological whole. The only geometry that satisfies the demands of economy and efficiency, while integrating multiple spatiotemporal scales in an interdependent and mutually informing manner, is fractal geometry – hence, "The Fractal Geometry of Nature" of Benoit Mandelbrot [155], hence allometric scaling laws in biology [154], hence disruption of fractal geometry, uncoupling of scales, and deterioration of long-range correlations in disease and aging [162-168].

Now, for comparison, let us consider how the conventional biological paradigm rooted in the frameworks of classical mechanics and equilibrium thermodynamics directs and informs research in biological sciences. It is fair to suggest that the elucidation of a novel metabolic pathway in a given organism, for example, would adhere to the following traditional formula. Individual enzymes of the metabolic pathway in question are purified to homogeneity and characterized in terms of their substrate specificities and catalytic activities. The metabolic pathway is *inferred*, drawn as a chart of sequential chemical conversions, and added to a growing integrated scheme of interlocked metabolic pathways. The pathway may be reconstituted with purified enzymes and shown to perform *in vitro*. Some or all of the metabolic intermediates may be detected and characterized. The presence of active enzymes of a given pathway in cell lysates is taken as proof that enzymes perform *in vivo *in the same way as *in vitro*. It is typical to repeat in other organisms assays for rigorously characterized enzymes of *E. coli *and, given positive results, to assume that the tested organisms have a metabolic pathway identical to that of *E. coli*. The presence of genes encoding for homologous enzymes in other organisms is often taken as proof that the pathway operates in the same way in all other organisms – "What is true for *E. coli *is true for the elephant" (Jacques Monod).

Obviously, by applying the same protocol and interpretations (which proved to be successful!) to all other metabolic pathways, a research community would inevitably, and very soon, come to see the cell as a well-mixed bag of reagents where diffusion- and concentration-driven enzymatic reactions take place. This image is then incorporated into textbooks, so that next generations of researchers learn it as a given and as a default. Any experimental evidence demonstrating, for example, reversible interactions between metabolic enzymes and structural proteins and membranes or channeling of metabolic intermediates would be naturally and inevitably treated either as artifacts or curious exceptions, which do not add much to or, worse, are inconsistent with the already "clear" textbook picture of metabolism and are thus not worthy of research efforts and funds and perhaps even one's attention. To treat them in any other way would be to go against such basic principles of rationality as the parsimony principle and the principle of least effort, not to mention career considerations in an environment in which conformity to peer review is an institutionalized pre-requisite of one's existence. The cell as a well-mixed biochemical reactor becomes a "square" fact, obvious to everyone (see Fig. 4). It becomes the reality. Once such a "reality" has crystallized as a structure in the minds of researchers and educators, it becomes an unconscious theoretical framework, a paradigm, a conventional wisdom, which directs and filters the experience of the research community as a whole and defines which methods, questions, and interpretations are legitimate and which are not, which projects and ideas are valuable and which are dispensable. For "...people most approve of what they best understand. ... Therefore, we adhere, as though to a raft, to those ideas that represent our understanding. This is a prime manifestation of vested interest. For a vested interest in understanding is more preciously guarded than any other treasure. It is why men react, not infrequently with something akin to religious passion, to the defense of what they have so laboriously learned." (J.K. Galbraith, from "The Concept of Conventional Wisdom" [172]). It should be emphasized that the established paradigm makes the research community blind to not just a few disparate facts, but to a large and continuously expanding fabric of experience comprising interconnected and interdependent observations, facts, ideas, and theories, which are consistent among themselves, but are inconsistent with and challenge the conventional image. It is of little surprise then that biochemical evidence indicating the existence of intracellular organization and circulation [93,94,118,123,134], direct microscopic visualization of elaborate cytoplasmic organization [130-132], experimental evidence suggesting compartmentalization of metabolism and substrate channeling [80,81,95,96], the flow theory of metabolism [133], alternative physical and physicochemical theories of the cell and intracellular organization and transport [120,122,173], and other related studies and theories [64,157,174,175] are suppressed or ignored (each to a different degree, of course, depending on how far a given fact/theory departs away from the convention and how strongly it challenges the accepted belief) in the mainstream literature and discourse, which, by necessity, are focused at any given moment on the elaboration and perpetuation of the conventional wisdom.

In order to break free from and move beyond the inadequate and stifling structure of the convention and thus to continue to grow in wealth, intelligence, and influence, on both the personal scale and the scale of society, one needs only to realize that classical physics and its associated worldview are not the end, but the beginning. And the fact that the beginning has been such a dramatic success does not mean we should stop right where we started. In fact, as empirical laws of nonequilibrium thermodynamics indicate and all of human history confirms, we have little choice: we either move forward and beyond the established structures, both in our minds and in our society, or we age and degrade.

## Competing interests

The author declares that he has no competing interests.

## Authors' contributions

AK is the sole author of this paper and is responsible for developing the concepts and for writing and revising the manuscript.
